# External Magnetization-Based Elasto-Magnetic Sensing for Tension-Change Estimation in Prestressed Concrete Tendons

**DOI:** 10.3390/s26217048

**Published:** 2026-11-07

**Authors:** Sehwan Park, Junkyeong Kim, Minkyo Youm, Seunghee Park

**Affiliations:** 1 AI Urban Disaster Prevention Research Center, Advanced Institute of Convergence Technology (AICT), Suwon 16229, Republic of Korea; sehwan0721@gmail.com; 2 Shutagen Company Ltd., Ulsan 44481, Republic of Korea; jkkim@shutagen.com; 3 School of Civil, Architectural and Environmental System Engineering, Sungkyunkwan University, Suwon 16419, Republic of Korea

**Keywords:** elasto-magnetic sensing, external magnetization, prestressed concrete, tendon tension, Hall sensor array, magnetic field measurement, change detection, structural health monitoring

## Abstract

Residual tension in tendons embedded in prestressed concrete girders cannot be measured with conventional elasto-magnetic sensors, because those sensors encircle the strand and must be installed before casting. This article evaluates a system that magnetizes an embedded tendon from outside the member and estimates the change in its total anchorage force from the local flux density. Three ferrite-core solenoids of 100, 200 and 300 mm outer diameter are placed on a common axis within a 300 mm envelope, and a board carrying 25 Hall and six temperature elements sits between the magnetizer and the concrete surface. Tests used one 2 m specimen containing twelve 12.7 mm strands in the target duct, at the nominal 80 mm embedment and 300 mm stirrup spacing; four loading–unloading rounds gave 40 acquired states. Estimating absolute tension failed to generalize across rounds for every regression model examined, the error exceeding the spread of the reference itself. A within-session difference representation gave a cross-validated coefficient of determination of 0.942 and a root-mean-square error of 169.0 kN, 13.5% of the 1252 kN full-scale reference. The binding constraint is therefore the signal representation rather than the regressor. Transfer across specimens, preservation of a reference between measurement epochs, and detection of small losses remain unverified.

## 1. Introduction

Prestressed concrete (PSC) bridges are widely used in road and civil infrastructure because prestressing compensates for the low tensile resistance of concrete and enables structurally efficient long spans [1,2]. The tendon is the element that introduces this compressive stress, suppresses cracking, and controls deflection under service load. Long-term performance therefore depends on whether the tension introduced at construction is retained close to its design level. With increasing service life, however, residual tension decreases progressively through elastic shortening, creep, shrinkage, relaxation, anchorage set, friction, and corrosion, and the resulting loss degrades crack resistance, deflection control, and the overall safety margin [2,3,4].

Damage that governs this degradation is not visible from the surface. Reinforcement corrosion, duct defects, grout voids, and section loss of the tendon develop inside the concrete [3,5], whereas routine inspection relies mainly on visual survey, crack mapping, and deflection checks, whose reliability depends on inspector experience, accessibility, and damage visibility [5,6]. Because internal tendons are embedded and cannot be observed directly, external inspection alone cannot detect tension loss at an early stage [2,3].

Several non-destructive testing (NDT) techniques address parts of this problem. Vibration-based methods infer prestress loss from changes in natural frequency, but the dynamic properties are weakly sensitive to tension and are confounded by boundary and environmental effects [1]. Acoustic emission detects strand fracture in real time, yet captures only events that have already occurred [7]. Impact-echo and ultrasonic methods locate grout voids in ducts but cannot quantify tension [8]. Magnetic flux leakage (MFL) is effective for section loss and fracture [9,10], but suffers from interference by surrounding reinforcement, limited inspection depth, and a formulation that targets the presence of a defect rather than the tension itself [11].

Work of the last few years has addressed the two difficulties that bear most directly on the present problem. The interference of ordinary reinforcement with magnetic measurement in concrete has been characterized as a function of the applied magnetization level, and the contribution of the bars has been separated from the measured profile by decomposition of its amplitude, correlation and offset components [12,13]; the relation between leakage-field variation and non-uniform section loss has likewise been quantified for corroded bars [14]. In parallel, magnetoelastic tension sensing itself has advanced through resonance enhancement, which raises sensitivity and reduces the influence of stress history [15]; recent reviews place these developments within the wider problem of effective-prestress evaluation [16], and magnetic flux-based assessment of post-tensioned tendons has been consolidated into inspection practice [17]. All of this work nonetheless presumes either a sensor on or around the strand, or a target that is itself the reinforcement rather than a tendon beneath it.

Tension measurement based on the elasto-magnetic (EM) effect, in which the permeability of a ferromagnetic material varies with stress, has been demonstrated on stay cables and external tendons [18,19,20,21]. Conventional EM sensors are through-type: a solenoid encircles the strand, so the sensor must be embedded during construction or installed where the tendon is exposed [21]. For an in-service bridge on which no sensor was pre-installed, the method is therefore inapplicable unless the concrete is cored and the reinforcement exposed. Recent work on machine-learning-based tension estimation [21], magnetic hysteresis characterization [22], and temperature compensation [23] has improved EM sensing accuracy, but assumes the same embedded or through-type configuration, so applicability to existing structures remains unresolved.

The external-magnetization concept, in which the tendon is magnetized without contact from the surface of the member, was investigated by the authors in [24] through analytical design, finite-element analysis and experimental implementation. The present article examines the particular magnetizer and multichannel Hall configuration described here, and the dependence of tension estimation on the representation of the measured signal. The contributions are as follows.

1. An implemented triple concentric ferrite-core solenoid magnetizer within a 300 mm planar envelope, with the calculated axial field of that arrangement compared against a single coil and a coplanar set of coils at a stated excitation.
2. A 25-channel Hall-sensor array whose radii were selected from strong-field and spatial-gradient metrics, with six-zone thermal instrumentation of the core and the printed circuit board (PCB).
3. A supervised channel-screening criterion that ranks channels from reference tension changes without assuming a channel count or array geometry, and that operates independently of the estimation algorithm.
4. A magnetomechanical finite-element model in which the strand is represented by a Jiles–Atherton material whose saturation magnetostriction was identified from tensioning measurements on a single strand, used as a qualitative interpretation of how an externally applied field couples to a strand lying perpendicular to the magnetizer axis.
5. Experimental evidence, on the tested configuration, that the dominant constraint on estimation is the signal representation rather than the regression model, and a within-session difference formulation that recovers prediction on tensioning rounds not used for training.

What the present article adds is therefore a matter of configuration and analysis rather than of priority. Table 1 sets the two side by side, following a comparison of the full text of [24] with the present manuscript.

The analysis reported here addresses three questions that follow from this configuration: how much of the measured Hall voltage is set by the measurement session rather than by the load; whether channels can be ranked from reference tension changes instead of from array geometry; and whether tension is better estimated from absolute voltages or from differences between two states acquired in the same session. These are evaluated on one laboratory specimen under cross-validation in which a complete tensioning round is withheld, and they do not establish transfer to a member whose tension is unknown. No figure, photograph or measured dataset of [24] is reproduced here. The accuracy reported in that study is referred to the coefficient of determination of a quadratic regression and is not commensurable with the root-mean-square error in percent of full scale reported below; the two are not compared.

Section 2 describes the elasto-magnetic effect, the external-magnetization requirement, the concentric solenoid array, the sensor placement metrics, the magnetomechanical finite-element model, and the direction in which the embedded strand is magnetized. Section 3 describes the fabricated system, the laboratory tensioning program and the treatment of self-heating. Section 4 reports the channel response, the failure of absolute estimation and the change-detection results. Section 5 concludes.

## 2. Sensing Principle and Array Design

### 2.1. Elasto-Magnetic Effect

The magnetization of a ferromagnetic material varies with its stress state, a coupling known as the elasto-magnetic or inverse magnetostrictive (Villari) effect [25,26]. Under an applied field *H*, the flux density inside the material is

(1) $$B=\mu_{0}\left(H+M\right)=\mu_{0}\mu_{r}H$$

where $\mu_{0}$ is the permeability of free space, *M* is the magnetization and $\mu_{r}$ is the relative permeability. Under a tensile stress σ the magnetic domains realign toward the stress axis, and the magnetoelastic energy is

(2) $$E_{\mathrm{me}}=\frac{3}{2}\;\lambda_{s}\;\sigma \sin^{2}\theta$$

with $\lambda_{s}$ the saturation magnetostriction and θ the angle between the easy axis and the stress axis [25,27].

Equations (1) and (2) are connected through the energy balance of the domain structure. Adding the magnetoelastic energy of (2) to the Zeeman energy and requiring the magnetization to settle at the minimum of the total energy is equivalent to retaining the Zeeman form alone and replacing the applied field by an effective field that carries the stress term [27],

(3) $$H_{\sigma }=\frac{3\sigma }{2\mu_{0}}\frac{d\lambda }{dM},\;H_{e}=H+\alpha M+H_{\sigma }$$

where α is the interdomain coupling coefficient. Stress therefore translates the anhysteretic curve along the field axis: at a fixed applied field *H* the magnetization *M* reached by the material changes, and with it the relative permeability $\mu_{r}$ that appears in (1). This is the mechanism that makes the flux density around a magnetized tendon a function of its tension [19,24].

The direction of the shift follows the sign of $\lambda_{s}$, and for prestressing strand that sign must be established rather than assumed. High strength steel is above the Villari reversal at the field levels applied here, so its saturation magnetostriction is negative and tension lowers the magnetization reached under a given applied field [25,26]. Tensioning a single ⌀12.7 mm strand inside a through-type coil confirmed this directly: the peak electromotive force of the secondary coil fell monotonically as the load was raised. Matching the finite-element model of Section 2.5 to those measurements located $\lambda_{s}\approx -1.4$ ppm, which is the value used throughout this article.

### 2.2. External Magnetization Requirement

Two requirements define the design problem. First, at the section of maximum eccentricity, which is the measurement target, the tendon lies approximately 8 to 10 cm below the accessible surface, so the external field must be strong enough at that standoff to change the magnetization state of the tendon measurably. Second, the reinforcement layout of a real PSC girder and the space available for installation limit the planar dimension of the magnetizer to about 300 mm, while field application requires reach up to about 30 cm. Because the field in free space decays rapidly with distance from the source, these two requirements reduce to maximizing reach from a bounded volume [24].

### 2.3. Concentric Solenoid Array

For a circular current loop of radius *R*, the on-axis flux density is proportional to $R^{2}{\left(R^{2}+z^{2}\right)}^{-3/2}$. A small radius therefore yields higher flux per unit current in the near field, whereas a large radius sustains the field at depth because the decay approaches $z^{-3}$. Superposition along the axis for a finite solenoid of winding density *n* and winding length *L* gives the closed-form on-axis expression reported in [28,29]; the off-axis field is expressed through elliptic integrals [28,30], and the three-dimensional distribution used here was obtained by applying the formulation derived in [24] to a multi-coil configuration. The derivations are not reproduced.

The radius–distance trade-off is resolved by placing solenoids of different radii on a common axis. By linear superposition, *N* concentric coils of radius $R_{i}$ and winding density $n_{i}$ produce an axial flux density

(4) $$B_{z}\left(z\right)=\sum_{i=1}^{N}\frac{\mu_{0}n_{i}I}{2}\left[f^{+}\;\left(R_{i},z\right)-f^{-}\;\left(R_{i},z\right)\right]$$

where $f^{\pm }$ are the two end terms of the finite-solenoid solution,

(5) $$f^{\pm }\;\left(R_{i},z\right)=\frac{z\pm L_{i}/2}{\sqrt{{\left(z\pm L_{i}/2\right)}^{2}+R_{i}^{2}}}$$

in which $L_{i}$ is the winding length of coil *i* and *z* is measured from the mid-plane of the winding along the common axis, the form $z\pm L_{i}/2$ placing the two end faces at $\pm L_{i}/2$. Because $L_{i}=100$ mm for all three coils, the end faces lie at z=±50 mm, and the plane quoted below as z=8 cm is therefore 30 mm beyond the upper end face of the windings, not 8 cm from it. The distinction matters: read instead as a distance from the coil face, the same plane would correspond to z=13 cm, where the computed on-axis flux density is 8.71 mT rather than 18.23 mT, a factor of 2.1. This analytical coordinate is also distinct from the embedment of the duct below the measurement surface and from the lift-off between the underside of the magnetizer and that surface; converting between them requires the installed geometry, and the embedment figure should not be read directly as a value of *z*.

**Figure 1:**
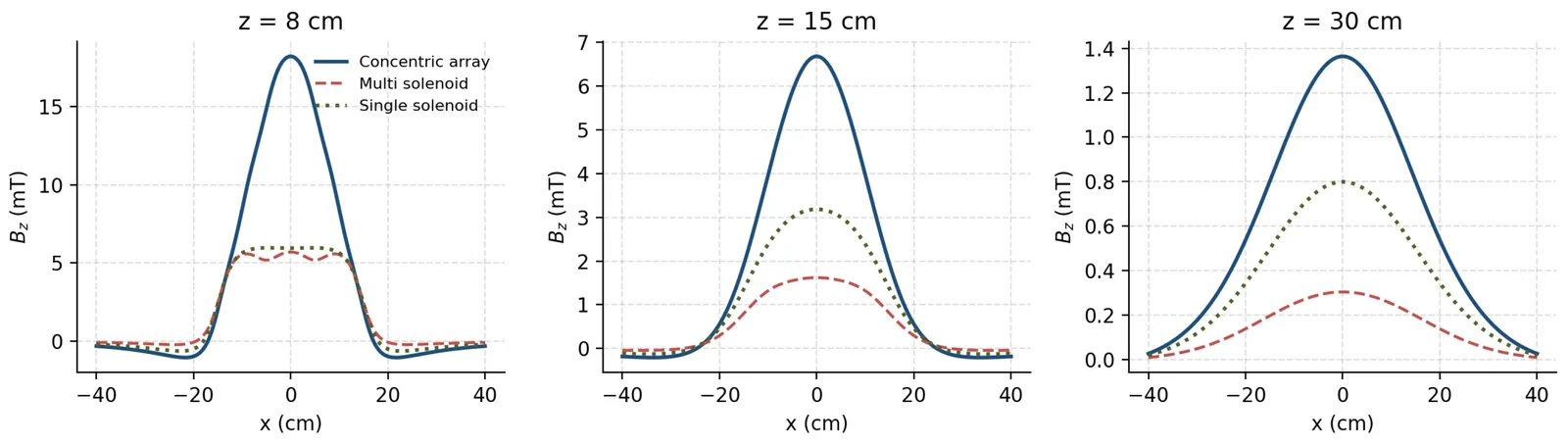
Axial flux density for the three coil configurations on the y=0 section, evaluated with (4): single solenoid (⌀300 mm), multiple solenoid (⌀100 mm × 3) and concentric array (⌀100/200/300 mm), at z=8, 15 and 30 cm. Here and throughout, *z* is measured from the mid-plane of the windings, as in (4), and not from the end face of the magnetizer; the end face lies at z=5 cm, so z=8 cm is 3 cm beyond it. Read against the end face instead, the value quoted below at z=8 cm would differ by a factor of about 2.1.

**Figure 2:**
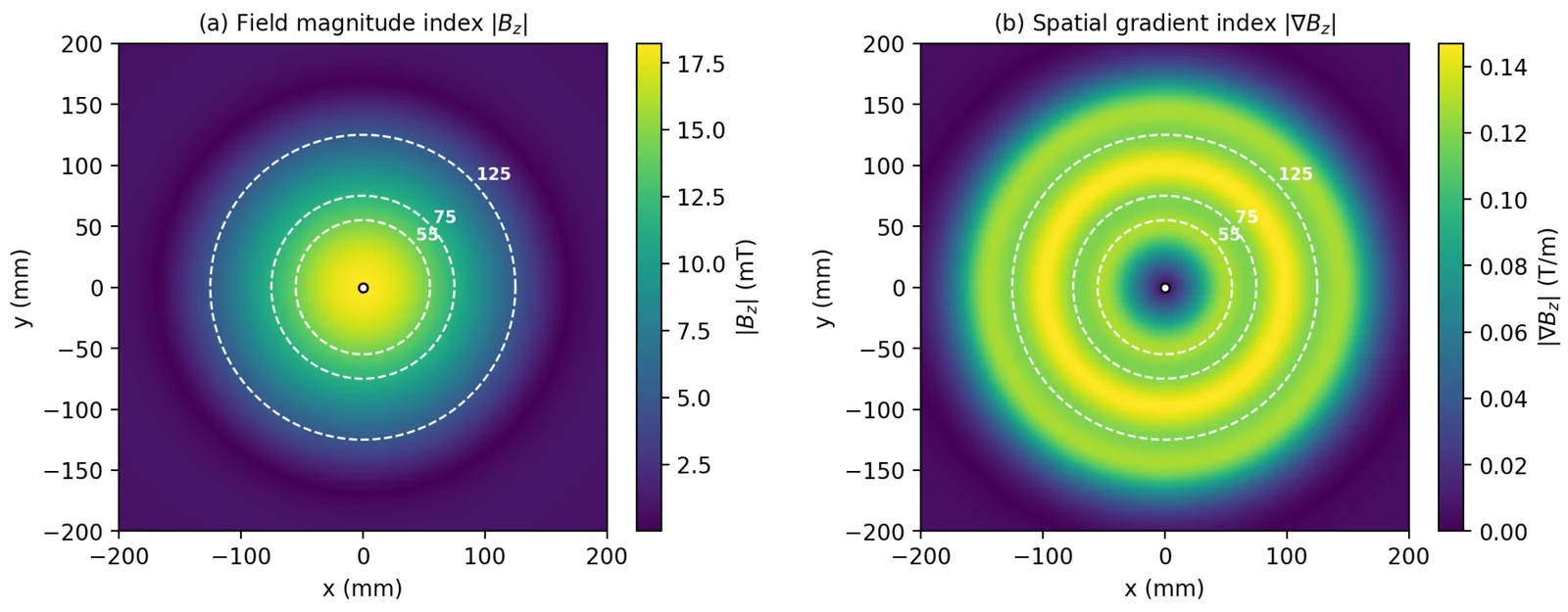
Placement metrics on the evaluation plane z=8 cm of Figure 1, that is 30 mm below the underside of the magnetizer, and the resulting Hall-sensor radii: (**a**) strong-field metric $|B_{z}|$; (**b**) spatial-gradient metric $|\nabla B_{z}|$. Section 2.3 gives the coordinate convention and the field at the tendon plane. The radii selected from these metrics do not depend on the choice of plane over the range of interest: between z=8 and z=13 cm the radius of maximum $|\nabla B_{z}|$ moves from 98 to 104 mm and the ordering of both metrics across the four installed radii is unchanged.

**Figure 3:**
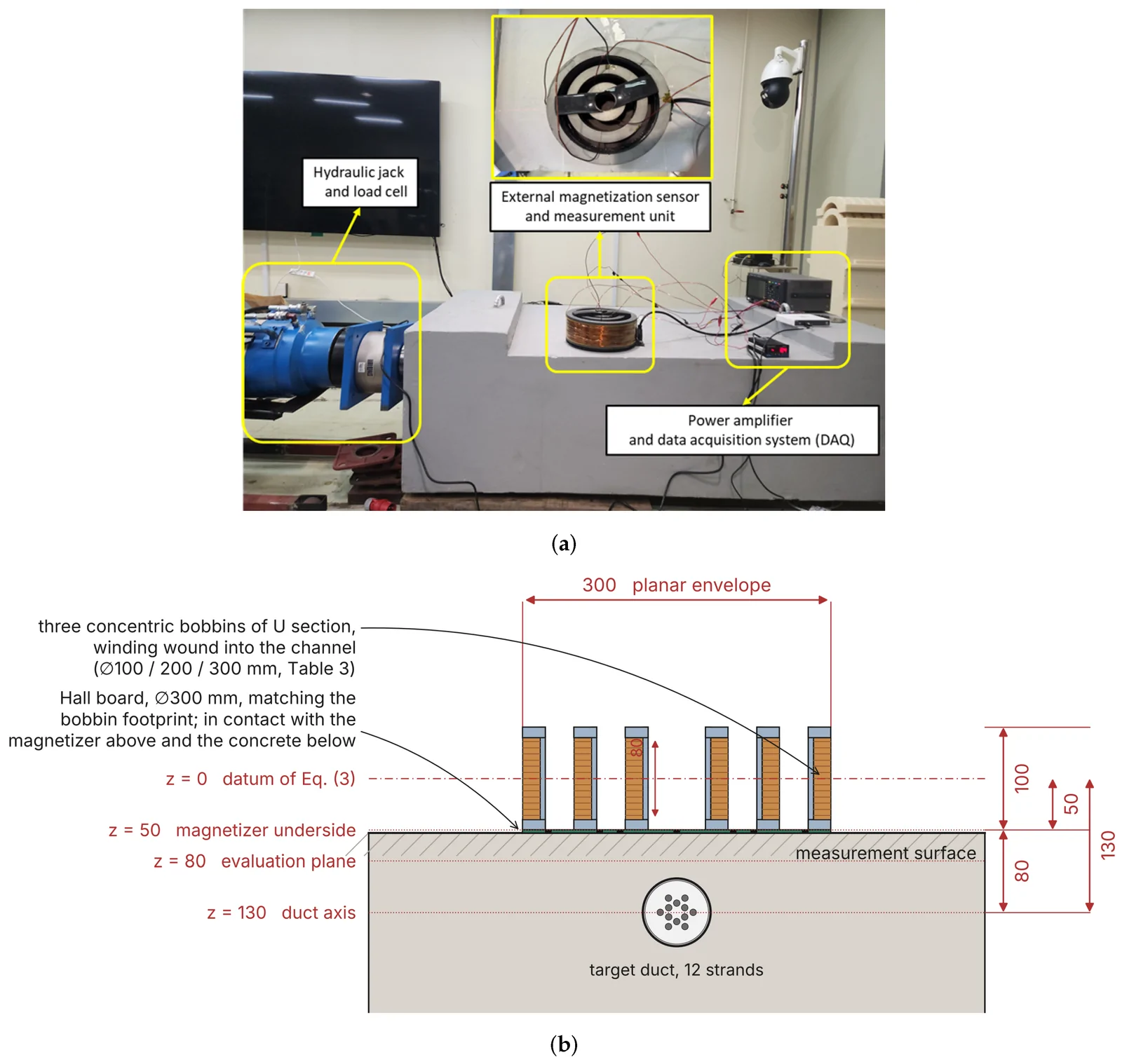
Test configuration and installed geometry: (**a**) tensioning test using a hydraulic jack and a hollow load cell; (**b**) vertical section through the magnetizer axis, with the reference points of the three distances used in this article. The datum z=0 is the mid-plane of the windings, as in (4); the underside of the magnetizer is at z=50 mm; the duct axis is 80 mm below that underside, at z=130 mm. The plane z=80 mm on which Figure 1 and Figure 2 are evaluated lies inside the concrete and carries no instrument.

One consequence of this convention must be stated, because the evaluation plane was chosen to represent the depth of the tendon and does not do so. The target standoff of Section 2.3 is referred to the underside of the magnetizer, and in the installed geometry of Section 3.4 the duct axis lies 80 mm below that face, which is z=13 cm in the coordinate of (4). The plane z=8 cm on which Figure 1 and Figure 2 are evaluated lies 30 mm below the face instead. The on-axis flux density of the concentric array at the tendon is therefore 8.71 mT, not the 18.23 mT quoted on the evaluation plane. The comparison between the three configurations is unaffected, since all three are evaluated on the same plane and the concentric array retains a factor of 2.26 over the single coil at z=13 cm against 3.06 at z=8 cm; what the evaluation plane does not provide is the absolute field available at the tendon, which is the value given here. Figure 3b gives the three reference points with their dimensions.

One further qualification applies to every flux density quoted in this section. Both the analytical expression (4) and the finite-element model of Section 2.5 represent the magnetizer by its windings alone: the ferrite cores of Table 2 are not included in either. The computed values are therefore those of an air-cored arrangement of the same geometry and excitation, and they understate the field produced by the device as built. They are used here to compare configurations, which the omission affects equally, and not as predictions of the absolute field at the tendon. Off the axis the same superposition is evaluated with the elliptic-integral form of [28,30]. The arrangement combines near-field concentration from the small coil with far-field persistence from the large coil, and makes the three fields add constructively about a common axis. Equation (4) was evaluated for radii of 50, 100 and 150 mm with $n_{1}=n_{2}=2115$ turns/m and $n_{3}=2065$ turns/m. These densities are the turn counts of Table 2 referred to $L_{i}=100$ mm, the total core height, and the same $L_{i}$ is used in (5); the effective winding height of approximately 80 mm quoted in Section 3.2 describes the occupied height of the conductor and is not the length used in the field model.

Figure 1 compares the axial flux density of the three candidate configurations on the measurement planes. On the axis at z=8 cm the concentric array reaches 18.23 mT against 5.95 mT for the single coil and 5.70 mT for the coplanar set, that is approximately 3.1 and 3.2 times, respectively, and approximately 2.1 and 1.7 times that of the single solenoid at z=15 and 30 cm. The comparison with the single coil does not isolate the effect of the arrangement, because the concentric array also carries 3.05 times the ampere-turns of that coil; the 3.1-fold ratio at z=8 cm is accounted for almost entirely by the excitation, and referred to ampere-turns the single large coil is in fact the more effective of the two at z=15 and 30 cm, where the concentric array returns 0.69 and 0.56 of its field per ampere-turn. In the radial direction, the multiple-solenoid configuration disperses the central flux through cancellation between coils, whereas the concentric array forms a single maximum on the axis, which is the distribution required for magnetizing a tendon located on that axis.

Because the three configurations differ in more than one respect, the basis of the comparison is stated explicitly. All three were evaluated at the same drive current of 10 A per coil, the same winding length of 100 mm, the same core material, and within the same 300 mm planar envelope and 100 mm height. The single-solenoid case is one coil of 300 mm outer diameter; the multiple-solenoid case is three coils of 100 mm outer diameter placed side by side on one plane; the concentric case is the three coils of Table 2 nested on a common axis. The concentric and the multiple configurations therefore carry essentially the same total magnetomotive force, 6295 and 6345 A-turns, while the single configuration carries 2065 A-turns. The concentric-versus-multiple comparison is the one that isolates the effect of the arrangement at equal excitation, and it is the comparison in which the concentric array gains a factor of 3.2 at z=8 cm, rising to about 4.1 and 4.5 at z=15 and 30 cm.

Two distinct effects are therefore present and should not be conflated. Against the coplanar set, at matched ampere-turns, the gain is attributable to the arrangement: coils placed side by side are not coaxial and their contributions partially cancel off the common axis, whereas nesting makes the three contributions add about one axis. Against the single large coil, the gain is attributable to excitation rather than to geometry, because nesting is what permits three independently driven windings, each with its own ferrite core, to occupy a 300 mm outer diameter and a 100 mm height that a single annular winding leaves largely empty. Raising the turn count of a single winding to the same total at a fixed wire gauge would increase the radial build beyond the envelope, and reducing the gauge to preserve the build would raise the resistance and the dissipation at the same current. The nested geometry is thus a way of placing more ampere-turns, and a single on-axis maximum, inside a fixed planar envelope. It is not claimed here to be the optimum at equal input power: the configurations were evaluated at a common 10 A per coil, not at matched power, and no equal-power optimization was carried out.

One property of this field bears on the measurement rather than on the design. On the evaluation plane the on-axis flux density falls by about 15.7% per centimeter of additional distance along the axis, and at the tendon plane by about 13.5%, so the figure is not sensitive to which of the two is taken. A difference of one centimeter in how the assembly is seated therefore changes the field at the elements by roughly a sixth. This is relevant to the common-mode term identified in Section 4.2, although it does not by itself account for it: the between-session coefficient of variation of the fitted slope, 23.3%, would correspond to a standoff difference of about 1.5 cm, which is larger than a laboratory fixture of this kind would plausibly vary. Standoff is therefore one contributor among several rather than the explanation, and the others, among them the residual magnetization of the previous cycle and the azimuthal seating of the array, are not separated here.

### 2.4. Hall-Sensor Placement Metrics

Flux change caused by tension is measured with Hall elements. For a semiconductor element of thickness *t* carrying a control current $I_{c}$, the Hall voltage produced by the flux density component $B_{z}$ normal to the element plane is

(6) $$V_{H}=\frac{I_{c}B_{z}}{n_{e}et}=S_{H}I_{c}B_{z}$$

where $n_{e}$ is the carrier density, *e* is the electron charge and $S_{H}$ is the sensitivity [31]. All elements were mounted so that they measure the component normal to the PCB plane, so the $B_{z}$ of (6) is used throughout the placement design and the subsequent analysis.

Because the flux distribution around the concentric array is spatially complex, sensors must be placed not simply where the field is strongest but where an adequate bias field coexists with a stable reflection of tension change in the output. Two metrics were therefore used. The strong-field metric $|B_{z}|$ identifies regions in which the element receives sufficient input for a small flux change to exceed its resolution. The spatial-gradient metric $|\nabla B_{z}|$ expresses the output sensitivity to small positional error; a large gradient favors differential signals across the distribution but also amplifies output variation caused by installation tolerance and tendon eccentricity.

Figure 2 shows both metrics on the placement plane. The strong-field metric is maximum on the axis and decreases monotonically with radius, whereas the spatial-gradient metric is near zero at the center and peaks locally near the winding radii. The two metrics are therefore opposed in radius and neither alone determines placement: using only the central region provides too little gradient information, while concentrating sensors where the gradient is maximum increases sensitivity to installation tolerance. Sensors were consequently distributed over radii that retain an adequate field level without a steep gradient, with a reference channel at the center, and symmetric positions were averaged to suppress installation bias.

Sensitivity to tension itself was examined separately, by comparing the computed change in $B_{z}$ between load steps in the coupled model of Section 2.5, with the qualifications on that model stated there. That comparison places the largest tension-induced change just beyond the footprint of the windings, farther from the axis than the outermost radius of the array as built. Two constraints prevent the elements from being moved there. The board must lie between the magnetizer and the concrete surface, because the same model shows the flux density at the sensor plane falling monotonically as that plane is raised, and the array is confined to the 300 mm planar envelope of the magnetizer set by the installation constraint above. Within that envelope the elements were distributed over the radii listed in Section 3.3, and the data-driven index of Section 4.2 then selects among the positions that are physically available rather than among the positions that would be ideal. The gap between the two is one reason why the achievable resolution is bounded as reported in Section 4.8, and closing it requires a magnetizer whose envelope and sensor plane are decoupled.

### 2.5. Magnetomechanical Finite-Element Model

The expressions above describe the source and take no account of the strand, the duct, or the dependence of the strand permeability on stress. A coupled finite-element model was therefore built in COMSOL Multiphysics v6.2 with the AC/DC and Solid Mechanics modules, in which the magnetizer, the duct and the strand are represented explicitly and the strand is loaded mechanically while it is magnetized. Table 3 summarizes the model.

The strand is represented by a Jiles–Atherton material rather than by a constant permeability, because its response to the applied field is strongly nonlinear, and its parameters were identified experimentally rather than taken from the literature. A small through-type EM sensor was wound for a single ⌀12.7 mm SWPC 7B strand and initial magnetization curves were acquired over a range of excitation amplitudes; the differential permeability $\mu_{d}/\mu_{0}$ ceased to increase and converged to unity near $1.6\times 10^{5}$ A/m, which gives a saturation magnetization of approximately $6.9\times 10^{5}$ A/m. The remaining parameters were obtained by sweeping *a*, *c* and *k* against the measured secondary-coil electromotive force, starting from published values for structural steel, a=470 A/m, c=0.0899, k=480 A/m and $\alpha =9.38\times 10^{-4}$ [32]. The sweep covered *a* from 350 to 700 A/m in eight values, *c* from 0.05 to 0.5 in seven, and *k* from 350 to 700 A/m in eight, that is 448 combinations in all. The saturation magnetostriction was identified separately by tensioning the same strand from 0 to 109.8 kN (11.2 tf) and minimizing the discrepancy between measured and computed electromotive force, which located $\lambda_{s}\approx -1.4$ ppm. The duct was modelled as 0.4 mm galvanized low-carbon steel, consistent with standard practice for prestressing duct enclosures, and the concrete was treated as magnetically equivalent to free space.

At the 10 A excitation used in the experiment the computed flux density reaches approximately 1.596 T in the duct wall and 0.463 T in the strand. Across the cross-section of the tendon the strands farthest from the measurement surface carry about two thirds of the flux density of those nearest it, which sets the depth over which the measurement is weighted within a single duct. The distance from the duct axis to the underside of the magnetizer was varied from 80 to 100 mm in 10 mm steps; the flux density at the sensor plane decreases monotonically over that range, which is why the Hall board is mounted between the magnetizer and the concrete surface rather than above the magnetizer.

The standing of this model should be stated before its results are used. The material parameters were identified from a single strand inside a through-type coil, a configuration in which the applied field is axial and the demagnetizing factor along the strand is negligible. Applying the identified law to a twelve-strand bundle inside a steel duct, magnetized from outside the member, assumes that it transfers to a different field distribution and a different demagnetizing condition. That transfer is not independently verified here: no measurement of the predicted local field or of its tension dependence inside the specimen is available, and the surrounding reinforcement is not represented in the geometry. The mesh and solver settings given in Table 3 are those of the two-dimensional axisymmetric model used to identify the material parameters; the corresponding statistics for the three-dimensional specimen model, and a formal mesh-convergence study, are not reported. The computed fields below are therefore used as a qualitative interpretation of the sensing mechanism and not as quantitative predictions of the measured signal.

### 2.6. Direction of Magnetization of the Embedded Strand

A through-type EM sensor magnetizes the strand along its own axis, where the demagnetizing factor is negligible. The magnetizer used here is mounted on the surface of the member with its axis normal to that surface, so that axis is perpendicular to the strand, and the direction in which the strand is actually magnetized therefore has to be established rather than assumed. The model of Section 2.5 was used for that purpose. Throughout this article *z* is the magnetizer axis, normal to the measurement surface, *x* is the longitudinal direction of the strand, and *y* is the remaining transverse direction.

Three results follow from the computed distribution, taken on a longitudinal and on a circumferential section through the array. First, $B_{z}$ is the largest component and dominates |B| on both sections, which is consistent with the on-axis design of Section 2.3 and is the reason the Hall elements are oriented to measure it; on the circumferential section the two components transverse to the magnetizer axis reach about half of $B_{z}$. Second, on the longitudinal section $B_{y}$, transverse to both the strand and the magnetizer axis, is negligible and shows no resolvable dependence on tension. Third, and the point that bears on the present question, $B_{x}$, the component along the strand, is not zero. It is antisymmetric about the mid-plane of the array, vanishing on that plane and reversing sign across it, and its magnitude varies monotonically with the applied tension, but the sense of that variation is not the same everywhere on the section. Section 2.1 establishes that $\lambda_{s}$ is negative at the field level applied here, so tension lowers $\mu_{r}$ and the strand carries less of the flux; inside the footprint of the windings the computed magnitude accordingly falls as tension is raised, while just outside the footprint, where the flux the strand no longer carries returns through the surrounding medium, it grows. These are one effect seen at two positions, and the growth outside the footprint is not a statement that tension raises the magnetization of the strand. Directly beneath the center of the array the flux lines diverge and the strand is magnetized weakly; the regions offset from the center along the strand are where it is magnetized most strongly, and the computed tension sensitivity is largest just outside the footprint of the windings.

The geometry of the strand amplifies this asymmetry. For a long slender ferromagnetic body the demagnetizing factor along the axis is close to zero while the transverse factor approaches one half, so the internal field that survives is predominantly longitudinal even when the applied field is not. The strand consequently acts as a longitudinal flux path beneath the magnetizer, drawing flux in on one side of the mid-plane and returning it on the other, and the reluctance of that path, $R=l/(\mu_{0}\mu_{r}A)$, carries the stress dependence of $\mu_{r}$ established in Section 2.1. On this reading the quantity interrogated is the same longitudinal stress-dependent permeability that a through-type sensor measures, with the longitudinal magnetization established by a different means. This is an interpretation drawn from the computed fields and from the slenderness of the strand, not a measured result; the internal magnetization of the strand was not observed, and the statement is not claimed to hold uniformly over the magnetized length.

What a Hall element receives is the superposition of the primary field of the magnetizer, which does not involve the strand and is fixed within a session at constant current, and the secondary field of the magnetized strand, which carries the tension information. The primary term is larger by a wide margin, which is why an absolute reading is governed by the magnetizer and its thermal state rather than by the tendon. Subtracting two states acquired in the same session removes the primary term identically and leaves the change in the secondary field. This offers a mechanism for the behavior reported in Section 4, where the change-detection representation succeeds and the absolute representation fails, although the measurements alone do not separate the two field contributions. It is consistent with the computed behavior of |B|, which decreases with tension inside the footprint of the array and increases outside it, a redistribution the source cannot produce on its own.

## 3. System Implementation and Experiment

### 3.1. System Configuration

The system comprises a magnetizer, a Hall-sensor measurement unit, a constant-current supply and a data acquisition (DAQ) unit. It is mounted on the external surface of the PSC member, applies an external field to the embedded tendon, and measures the local flux density change caused by tension change with a multi-point Hall array. The support structure of the magnetizer and the measurement unit was fabricated from plastic and non-magnetic fasteners to minimize distortion of the surrounding field.

### 3.2. Concentric Solenoid Magnetizer

Three ferrite-core solenoids of different diameter were placed on a common axis. The core material is a TPW33-series ferrite, selected for high initial permeability and high electrical resistivity. Outer diameters are 100, 200 and 300 mm, the total core height is 100 mm, and the effective winding height is approximately 80 mm. Windings use 2.0 mm PEW wire in six layers. Table 2 lists the measured specifications.

A direct current of 10 A was applied to each coil in the same magnetizing direction. The excitation is unipolar direct current; no pulsed or alternating waveform is used, and each coil is driven by its own channel of a four-channel programmable supply (Keysight E36441A) in constant-current mode, so that the three currents are set independently. The supply is rated to 25 A and its current accuracy is 0.1% of setting, with a constant-current ripple of 75 mA rms. Constant-current drive minimizes current ripple and output drift, which keeps the magnetizing condition uniform across repeated measurements. With the measured resistances of Table 2, the peak electrical power of the magnetizer is 211.3 W and the compliance voltages required are 3.4, 7.0 and 10.7 V. Current is applied only while a load step is being acquired and not continuously, so the average dissipation over a complete tensioning session is well below that peak.

Because the coils differ in resistance and because continuous current causes heating that alters both the magnetic properties of the core and the Hall output, the temperatures of the core and the PCB were measured simultaneously for subsequent correction.

### 3.3. Hall-Sensor and Temperature Measurement Unit

The measurement unit is a PCB installed between the magnetizer and the specimen surface. Twenty-five Hall elements are arranged symmetrically about the coil axis, comprising one central element and elements at radii of 55, 75 and 125 mm, all oriented to measure $B_{z}$ normal to the PCB plane. Table 4 gives the layout.

Table 5 gives the electrical specification of the measurement chain. The supplied circuit identifies the sensing elements as being of the DRV5055A4 family [33], operated from a 5 V supply, at which the nominal sensitivity is 12.5 mV/mT about a quiescent output of half the supply voltage. The full ordering suffix was not recovered and the sensitivity was not independently calibrated, so the manufacturer values are used as nominal figures. All 32 analogue channels are read by one 20-bit multifunction unit [34]. The 5 V rail of the Hall board is taken from the +5 V terminal of that same unit, which is specified at +4.87 to +5.22 V unloaded, +4.76 to +5.17 V under load, with a 280 mA current limit [34]. No separate regulator was interposed, and the rail was not recorded on a measurement channel. Its absolute tolerance, about ±4%, is therefore wide compared with the quantities of interest, and Section 4.6 examines what follows from that and bounds the term from the acquired data. The two noise figures available for the element agree: an input-referred density of 130 nT/$\sqrt{\mathrm{Hz}}$ over the 20 kHz sensing bandwidth corresponds to about 0.23 mV rms, or roughly 1.4 mV peak to peak, against the 1.5 mV peak-to-peak output noise quoted for the board.

Two consequences of this chain are used later. A tension-induced change of 2.3 mV at a channel corresponds to about 0.18 mT of flux density, and the quantization step of roughly 10 μV places that change some 230 steps above the resolution floor, so the converter does not limit the measurement. The absolute accuracy of 692 μV is, by contrast, about 30% of that change; it is dominated by gain and offset terms that are fixed within one acquisition session, which is one reason a difference taken within a session behaves differently from an absolute reading.

The absolute output of a single element is sensitive to tendon eccentricity, installation position and local field distortion, so elements were grouped by radius to allow radial response to be compared. Measurement quality depends on angular position as well as radius, however, so no group was excluded or prioritized in advance; all 25 channels were retained and channels were selected using the quality index defined in Section 4.2.

Zonal heating under direct current was measured with one ambient channel, three ferrite-core channels and three PCB channels, with the core and PCB sensors placed at central, inner and outer positions. The acquisition channel map is ambient temperature (Ch. 0), Hall elements (Ch. 1–25), core temperature (Ch. 26–28) and PCB temperature (Ch. 29–31); all channels were acquired synchronously.

### 3.4. Specimen and Tensioning Test

A 2 m uniaxial specimen reproducing the section of a PSC I-girder was used, with dimensions taken from the standard drawings of existing PSC beam bridges. Three ducts of 66 mm outer diameter run longitudinally, and the standoff between the measurement surface and the target tendon is approximately 8 to 10 cm, matching the distance at the section of maximum eccentricity. The target tendon consists of twelve 12.7 mm prestressing strands. The target duct was left ungrouted, so that the force in the tendon could be raised and released repeatedly over the four tensioning rounds; a bonded duct would not have permitted this. The consequence for the reference quantity is taken up in Section 4.9. The specimen was cast with more than one configuration so that the influence of the installation geometry could be examined: duct embedment depths of 80, 150 and 300 mm and shear reinforcement spacings of 300 and 150 mm are present in different regions of the member. The measurements reported in this article were all taken on the 80 mm embedment within the 300 mm stirrup spacing, which is the configuration nearest to the section of maximum eccentricity of a real girder; the remaining configurations were not measured in this work; Section 4.9 identifies them as accessible without new fabrication.

The magnetizer was centered within the region of 300 mm stirrup spacing to reduce interference from surrounding reinforcement, and its axis is normal to the measurement surface. The magnetizer axis is therefore perpendicular to the longitudinal axis of the tendon, and Section 2.6 establishes the consequence of that arrangement for the direction in which the strand is magnetized.

The lateral position of the assembly with respect to the tendon was treated as a design variable. In rounds 1 and 2 the axis was positioned to intersect the target tendon, so that the array was centered over the tendon in plan. In rounds 3 and 4 the magnetizer and the measurement board were displaced together by −50 mm and +50 mm perpendicular to the tendon axis and parallel to the measurement surface, respectively. The two parts remained rigidly coupled, so the field produced at each Hall element by the coils is unchanged by the displacement and only the geometry relative to the tendon differs. The four rounds therefore comprise two acquisitions at zero eccentricity and one at each of the two offsets, and Section 4.5 evaluates the two conditions separately.

The magnetizer was not removed between rounds 1 and 2. Those two rounds were acquired in succession with the assembly left in place, and only the magnetizing current and the load were cycled; rounds 3 and 4 followed the deliberate lateral displacements described above. No round therefore repeats an installation at the same nominal position, and the fixed-position figures of Section 4.5 measure reproducibility between acquisitions rather than between installations. The distinction is taken up in Section 4.9.

Figure 3b gives the installed geometry with its dimensions, and the reference points require a word because three different ones are used in this article and they are not interchangeable. The datum of the field model, Equation (4), is the mid-plane of the windings. The underside of the magnetizer lies one half winding length below it, at z=50 mm. The embedment of the duct is measured from the concrete surface. Because the Hall board is in contact with the magnetizer above and with the concrete below, the underside of the magnetizer and the measurement surface are separated only by the thickness of the board, which is small beside the 80 mm embedment. The duct axis therefore lies at z=130 mm, and the two statements made elsewhere in this article—80 to 100 mm from the underside of the magnetizer in Section 2.5, and 8 to 10 cm below the measurement surface in Section 2.3—describe the same distance to within that thickness.

The plane z=8 cm on which Figure 1 and Figure 2 are evaluated is not a surface of the assembly at all. It lies 30 mm below the underside of the magnetizer, inside the concrete, and carries no instrument. The submitted version of this article described it as the sensor plane. It is not: the Hall elements lie at the underside of the magnetizer and the tendon 80 mm below them. With that description corrected there is no discrepancy between the 30 mm standoff of the evaluation plane and the 80 to 100 mm duct distance of the finite-element model, because the two are referred to different points and only the second describes the experiment.

Tension was introduced with a hydraulic jack at the specimen end, and a 1961 kN (200 tf) hollow load cell measured the actual tension at each step. Hydraulic pressure was increased from 0 to approximately 380 bar in steps of about 100 bar and then reduced stepwise. The load-cell reading, not the converted hydraulic pressure, was used as the reference tension. Loading and unloading data were separated to account for magnetic and jack hysteresis. Throughout this article a *round* denotes one complete tensioning cycle and a *session* denotes the loading or the unloading sequence within a round, so each round contains two sessions. Figure 3 shows the configuration. At each step, the load was held at the target level, 10 A direct current was applied to each of the three coils, and the 25 Hall channels together with the core, PCB and ambient temperatures were acquired through the DAQ.

### 3.5. Preprocessing and Temperature Handling

Continuous direct current raises the temperature of the ferrite core and the PCB, and the Hall voltage can change at constant tension for two reasons: the permeability of the ferrite is temperature dependent, and the Hall element itself has a sensitivity temperature coefficient, quoted as 0.12%/°C for the DRV5055 family. The second can be evaluated without any field calculation, because the field-dependent part of a channel’s output is its measured standing output less the quiescent value $V_{\mathrm{CC}}/2$. Over the eight retained channels that quantity ranges from 27.9 to 545.8 mV, so the coefficient alone predicts a thermal response of 0.03 to 0.66 mV/°C, which contains the largest measured value of 0.54 mV/°C reported below; four of the eight channels predict between 0.31 and 0.66 mV/°C. The element therefore accounts for a substantial part of the measured response, the two contributions are not separated by the present measurement, and the response should not be read as a property of the ferrite alone. A separate no-load test was therefore run in which the magnetizing current was applied continuously and the temperature rise was measured stepwise; thermistor voltages were converted to degrees Celsius using the NTC characteristic. Rather than applying a scalar correction, the measured temperatures were included directly as covariates of the estimation model: the three core zone sensors, the three PCB zone sensors and the ambient sensor, seven temperature covariates in total. In the change-detection representation these covariates are differenced together with the Hall voltages, so that a differential sample carries the change in each of the eight retained channels and the change in each of the seven temperatures between the two states; they enter as first-order terms only.

**Figure 4:**
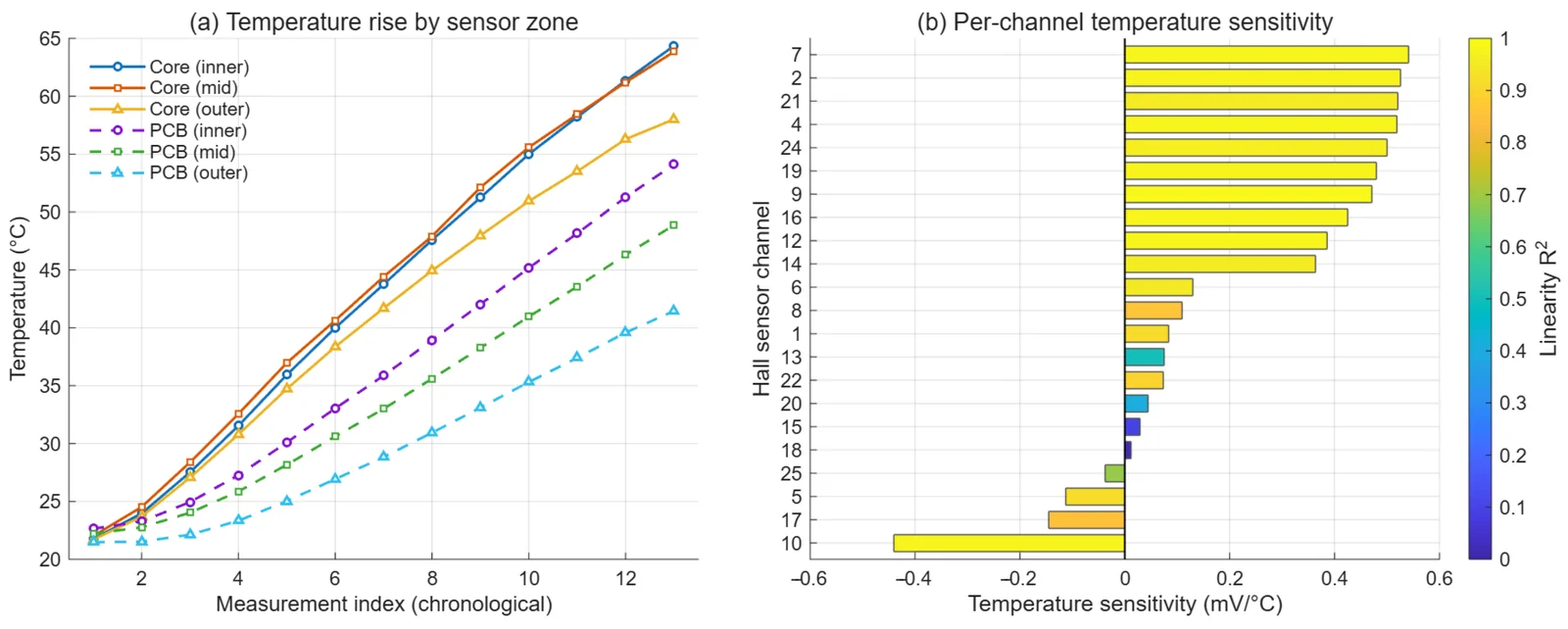
No-load thermal test: (**a**) temperature rise by zone; (**b**) the available per-channel temperature sensitivities, with bar color indicating linearity $R^{2}$. Panel (**b**) displays 22 channels: channels 3, 11 and 23 are absent from the retained output of this no-load test, which is a separate acquisition from the tensioning program, and no thermal sensitivity is implied for them. All 25 channels are present in the tensioning dataset and appear in Figure 5.

Two observations from the no-load test motivate this choice. First, as shown in Figure 4a, all zones rose from about 22 °C, with the core interior highest and the PCB exterior lowest, and the spread widened continuously; at the end of the measurement the core interior reached about 64 °C and the PCB exterior about 42 °C, a difference of about 22 °C at the same instant. A single temperature value therefore does not represent the thermal state of the assembly. Second, among the channels displayed, Figure 4b shows that the sensitivity of the Hall voltage to temperature ranges from about −0.44 to +0.54 mV/°C, that the outputs of some channels rise while others fall, and that the largest sensitivity exceeds the smallest by more than a factor of twenty in absolute value. A single coefficient applied uniformly is therefore invalid. Channels with high sensitivity showed coefficients of determination close to unity, whereas channels with near-zero sensitivity also showed low determination; this reflects negligible temperature dependence rather than poor measurement quality, since their outputs barely changed over a rise of about 42 °C. Because the relationship is close to linear over the measured range, the covariates were entered as first-order terms.

**Figure 5:**
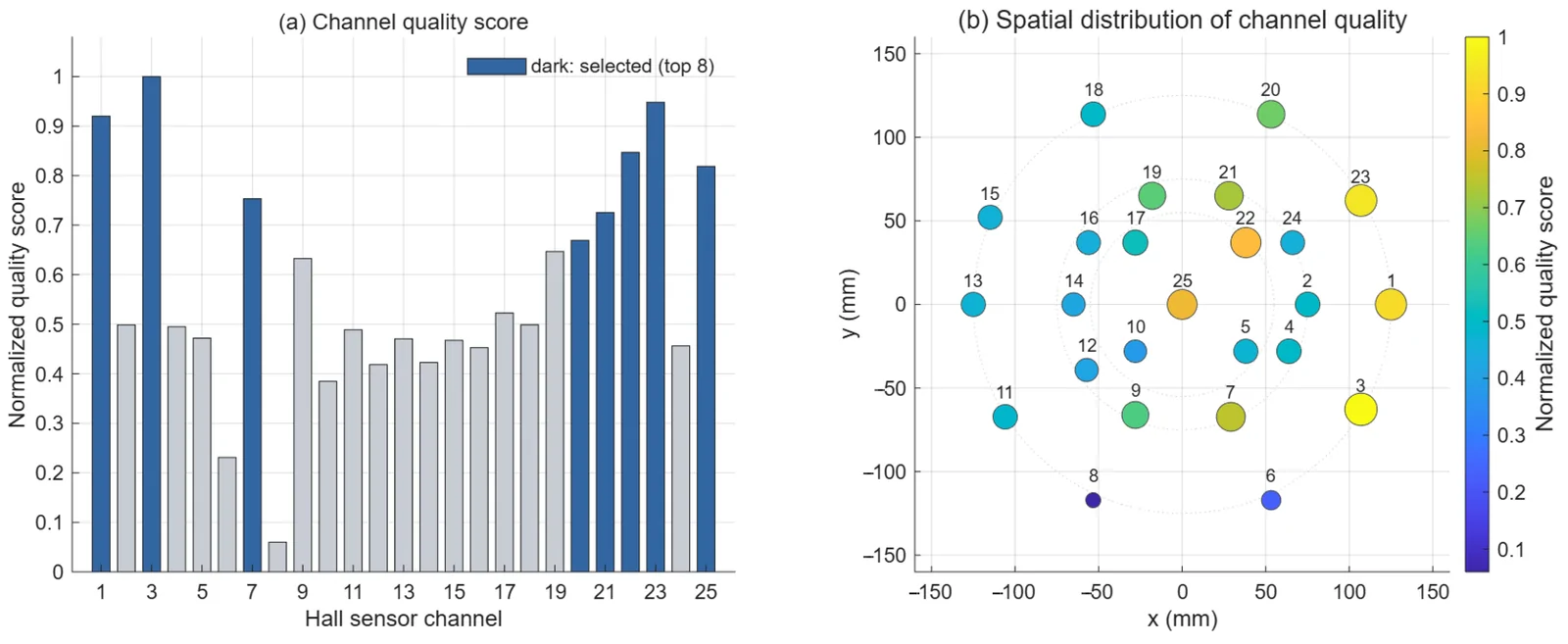
Automated channel selection based on the quality index of (7): (**a**) normalized index by channel, with the eight selected channels shown as dark bars and the seventeen channels not selected in grey; (**b**) spatial distribution of the index over the sensor plane, drawn at the radii and azimuths listed in Table 4. Both panels are evaluated over the complete tensioning dataset and illustrate the index itself; neither corresponds to a single fold. The selection entering the cross-validated results of Section 4.4 is recomputed inside each training fold, as Section 4.1 states.

These two observations concern the sensitivity of the channels to temperature, which is a property of the instrument. Whether that sensitivity matters for the estimate is a separate question, and Section 4.6 answers it by withholding the covariates: on this dataset they did not improve accuracy. The covariates are retained in the reported configuration because a field deployment would span a far wider thermal range than the laboratory, but their benefit under those conditions is not demonstrated here.

Because the six zone temperatures rise through the same heating process and are strongly correlated, no single zone was taken as representative. Session-to-session differences in residual magnetization and installation also shift the initial signal offset, so Section 4 evaluates estimation from absolute Hall voltages against estimation from the difference between two states within one session. Differencing removes an additive term only to the extent that it is common to the two states, so it addresses the offset of one session and not any difference between sessions, nor any change in sensitivity. Loading and unloading are kept in separate sessions to prevent mixing of hysteresis effects.

The magnetizing current was held at 10 A by constant-current control, so drift of the source is excluded, and the temperature dependence of the ferrite permeability is carried by the covariates above rather than by a separate conversion coefficient. This treatment holds for the quasi-static thermal state of laboratory testing. In long-term monitoring a seasonal difference between the reference and the re-measurement epoch introduces a temperature component into the differential signal; the covariates already accommodate that case in form, but the seasonal range differs in distribution from the self-heating range examined here.

## 4. Results and Discussion

### 4.1. Dataset and Evaluation Protocol

Four rounds of laboratory tensioning data were analyzed. Each round comprises five load steps under loading and five under unloading, that is two sessions, giving eight sessions and 40 measurement files in total, with reference tension from 0 to 1252 kN (0 to 127.7 tf). Each measurement acquired all 32 analogue channels at 100 kS/s for between 3.5 and 5.2 s, a median of 3.8 s, giving approximately $3.8\times 10^{5}$ samples per channel. The per-channel feature is the arithmetic mean over that window.

A zero-phase fourth-order Butterworth low-pass, applied forwards and backwards, precedes the averaging. Its corner was specified as 5 Hz against an assumed 2 kHz rate; at the 100 kS/s rate actually used this normalized corner corresponds to about 250 Hz. Neither value governs the feature, because the window mean over 3.8 s has an equivalent bandwidth near 0.13 Hz and is by far the narrower operation. The point is confirmed directly: repeating the entire cross-validation with the filter disabled reproduced the reported figures exactly, with an LSBoost RMSE of 17.23 tf in both cases and the three other models agreeing to 0.01 tf. The filter is therefore not a model parameter, and the accuracy reported below is not limited by electronic noise. The quoted peak-to-peak output noise of a Hall element, averaged over an acquisition window, falls below one microvolt against a tension-induced change of about 2.3 mV; what limits the measurement is the reproducibility of the installation, as Section 4.8 shows.

Because the objective is estimation on an aged bridge for which no prior information exists, generalization to data not used for training was adopted as the evaluation criterion, and leave-one-round-out cross-validation was applied: one round is held out in full, including both of its sessions, the model is trained on the remaining three rounds, and the held-out round is predicted, repeating over all four rounds. Holding out the whole round rather than a single session prevents the loading and unloading sequences of the same cycle from being split between training and validation. Channel selection and hyperparameter determination were performed inside the training folds only, using a nested leave-one-group-out loop, so no information from the validation round enters training. The eight highest-ranking channels of the 25 were retained in each fold.

The design variables of the tensioning program were the load level and the loading direction; the specimen geometry, the standoff and the strand configuration were held fixed, so the generalization examined here is across tensioning cycles rather than across members. Four rounds is a small number of independent folds, and the cross-validated figures below should be read as estimates from four folds rather than as converged population values. Section 4.9 states which variables remain unexplored and Section 2.5 reports what the finite-element model contributes on the ones that were not varied experimentally.

The quantitative metrics are the coefficient of determination $R^{2}$, the root-mean-square error (RMSE), the RMSE as a percentage of full scale (%FS), and the quantity 1.96RMSE, which is reported as a nominal error scale rather than as a validated minimum detectable change; the assumptions it rests on are examined in Section 4.8. Four regression models were compared to examine the effect of model complexity: ridge regression (Ridge), partial least squares regression (PLS), Gaussian process regression (GPR) and least-squares gradient boosting of regression trees (LSBoost).

### 4.2. Channel Quality Index and Automated Selection

Hall outputs changed stepwise with tension, reflecting the permeability change produced by the elasto-magnetic effect, but the magnitude of the response differed between channels and was governed by angular position as well as radius. This follows from the external-magnetization configuration, in which the flux distribution is set by the relative placement of the magnetizer and the tendon. Channel effectiveness is therefore not determined by array geometry alone but depends jointly on the eccentric position of the tendon within the duct and on the alignment of the magnetizer. Fixing a particular channel or radial group in advance would not remain valid when the target member or the installation condition changes.

A quality index that quantifies channel effectiveness directly from the measurement data was therefore defined. For channel *k*,

(7) $$Q_{k}=\frac{\left|\rho \;\left(\Delta V_{k},\Delta T\right)\right|}{\sigma_{k}}$$

where ρ is the correlation coefficient between the differential signal of the channel and the tension change, and $\sigma_{k}$ is the standard deviation of the residual of a linear fit of ΔT on $\Delta V_{k}$. With this regression direction $\sigma_{k}=\sigma_{\Delta T}\sqrt{1-\rho^{2}}$, in which $\sigma_{\Delta T}$ is common to all channels, so $Q_{k}$ carries the reciprocal of the tension unit, kN^−1^ here, and only the normalized score plotted in Figure 5a is dimensionless. The index therefore has no dependence on the voltage scale of a channel: it is a strictly increasing function of $\left|\rho \right|$ alone, the rank correlation between $Q_{k}$ and $\left|\rho \right|$ over the 25 channels being 1.000, and the normalization sets the units without altering the ordering. The numerator captures sensitivity and sign consistency with respect to tension change, and the denominator places the index on a tension scale, so a high index identifies a channel that reflects tension change strongly and consistently with low noise. The index makes no assumption about array geometry or channel count, so its definition carries over to any multichannel configuration; it has been evaluated here only on the present array, at the single embedment and stirrup spacing of Section 3.4 and over lateral eccentricities of 0 and ±50 mm, and transfer beyond those conditions is untested. It is also a supervised criterion: it is computed from reference tension changes and therefore requires measurements of known load. It selects channels without assuming where the good channels are, not without calibration.

Figure 5 shows the result for the present dataset. The index varied markedly between channels, and even among channels at the same radius the ratio of maximum to minimum reached approximately sixteen. Its spatial distribution was not random but oriented: the highest-ranking channels clustered toward one azimuth and the lowest values occurred in the opposite region. This directional gradient is attributed to asymmetry of the flux distribution caused by eccentric placement of the tendon within the duct, and supports the argument above that channel effectiveness depends on installation condition.

A threshold on the index itself was deliberately not used: $Q_{k}$ has no absolute scale and its distribution shifts from fold to fold, so a fixed numerical cut would admit a different number of channels in each fold and confound the comparison between representations and between models with a varying feature count. The count was instead held constant, and its value was examined by repeating the cross-validation with the number of retained channels *K* varied from 1 to 25 on the differenced Hall signals alone, with hyperparameters fixed. The RMSE of the boosted-tree model falls monotonically from 233.7 kN at K=1 to 146.3 kN at K=25, a reduction of 37%, so the multichannel array contributes materially rather than decoratively.

Retaining all 25 channels therefore gives a lower error than retaining eight, and the screening is not an accuracy gain. Its role is diagnostic: it identifies channels that respond weakly or inconsistently to tension, and it shows that the responsive channels are distributed across all four radial groups rather than confined to one, which is the result that bears on array design. The fixed count of eight was adopted to hold the feature dimension constant across folds and across the comparisons of Section 4.4, not because it maximizes accuracy, and the accuracy reported below is correspondingly conservative.

The shape of that reduction is more informative than its size. If the channels carried independent errors the RMSE would fall as $K^{-1/2}$. The fitted power-law exponent is −0.16 for the boosted-tree model and −0.26 for the linear one, both far shallower, and the data are better described by ${\mathrm{RMSE}}^{2}\left(K\right)=G+N/K$ with a floor *G* between about 128 and 157 kN that does not decrease with *K* at all. A substantial part of the error is therefore common to every channel. The excitation current, the standoff between the array and the tendon, and the azimuthal placement of the array are shared by all channels at a given installation and are plausible origins of such a common-mode term, which no increase in channel count can average away. That two estimators of different character give the same exponent indicates a property of the data rather than of the model.

K=8 was retained for the reported results. Using the full array would lower the RMSE to 146.3 kN, or 11.7%FS, a gain of 1.8%FS; the diminishing return above places that gain second order relative to the common-mode floor, and retaining a subset preserves the diagnostic role of the index in identifying degraded channels.

The retained set is not identical in every fold. Three channels were selected in all four folds, and fourteen of the twenty-five were selected at least once, so the index identifies a group of channels of comparable informativeness rather than a unique optimum. Accuracy was nevertheless stable across folds under this protocol, which indicates that the array carries redundancy against the degradation of an individual channel. The channels favoured for absolute estimation differ from those favoured for change detection, consistent with the two tasks rewarding different properties: stability of offset in the first case and sensitivity in the second.

One further check bears on what the index measures. Because the measurements were acquired in sequence, a channel that drifted monotonically through the program could in principle rank highly without responding to tension at all. Replacing the tension difference in (7) by the acquisition index gives correlations that do not exceed 0.13 for any of the eight selected channels, and 0.31 for any channel in the array, against 0.96 or above for the tension correlation of the selected set. The ranking therefore follows the tension signal rather than the order in which the measurements were taken.

The eight retained channels were distributed across all four radial groups: one central element, one at 55 mm, two at 75 mm and four at 125 mm. Several channels at the outermost radius, which had been expected to be the most sensitive to installation deviation, ranked among the best. Channel selection should therefore be based on measurement data rather than on geometric grouping of the array. Because the index is computed inside the training fold only, no information from the validation round enters the selection, and because the criterion is derived from data, the same procedure applies without prior labels from the held-out round when the array orientation, the tendon eccentricity or the member condition differs within this specimen. It does not follow that the procedure transfers to a member whose tension is unknown: both the screening and the model are developed from known-load data acquired on this specimen, and withholding the labels of one round tests transfer between rounds of that specimen rather than transfer to an uncalibrated member. The procedure operates independently of the estimation algorithm and is in that sense usable as a general signal-quality management step for multichannel magnetic measurement systems.

### 4.3. Limitations of Absolute Tension Estimation

Estimating tension directly from the absolute Hall voltage of each measurement was evaluated first. All four regression models returned $R^{2}<0$, that is, they failed to generalize to rounds not used for training; a negative $R^{2}$ means that prediction is worse than using the mean of the training data. The mode of failure differed between models. LSBoost and GPR saturated within the training range, so their RMSE remained at 42.0%FS and 53.2%FS, whereas Ridge and PLS extrapolated on validation inputs far outside the training range and diverged beyond 700%FS. No model generalized across rounds.

The cause is that residual magnetization of the tendon and the core differs between sessions, so both the offset and the sensitivity of the absolute Hall voltage vary. To quantify this, the Hall voltage of each channel was fitted as a linear function of tension for each session, and the between-session standard deviation of the intercept was compared with the signal swing produced by tension change within a session. For the highest-quality channels, the between-session standard deviation of the intercept was approximately 3.8 to 7.4 times the tension-induced swing, with a median of 5.9. The session-variation component of the absolute signal is therefore much larger than the tension component, and the load information is buried. This figure, and the slope variation reported in Section 4.4, quantify the combined effect of everything that differs between sessions: the residual magnetization left by the previous magnetizing cycle, the thermal state of the core, and any difference in how the magnetizer was seated. Of these, the lateral position of the array was varied deliberately, and Section 4.5 reports its effect separately; the residual magnetization and the thermal state of the core were not controlled independently and are not separated here.

**Figure 6:**
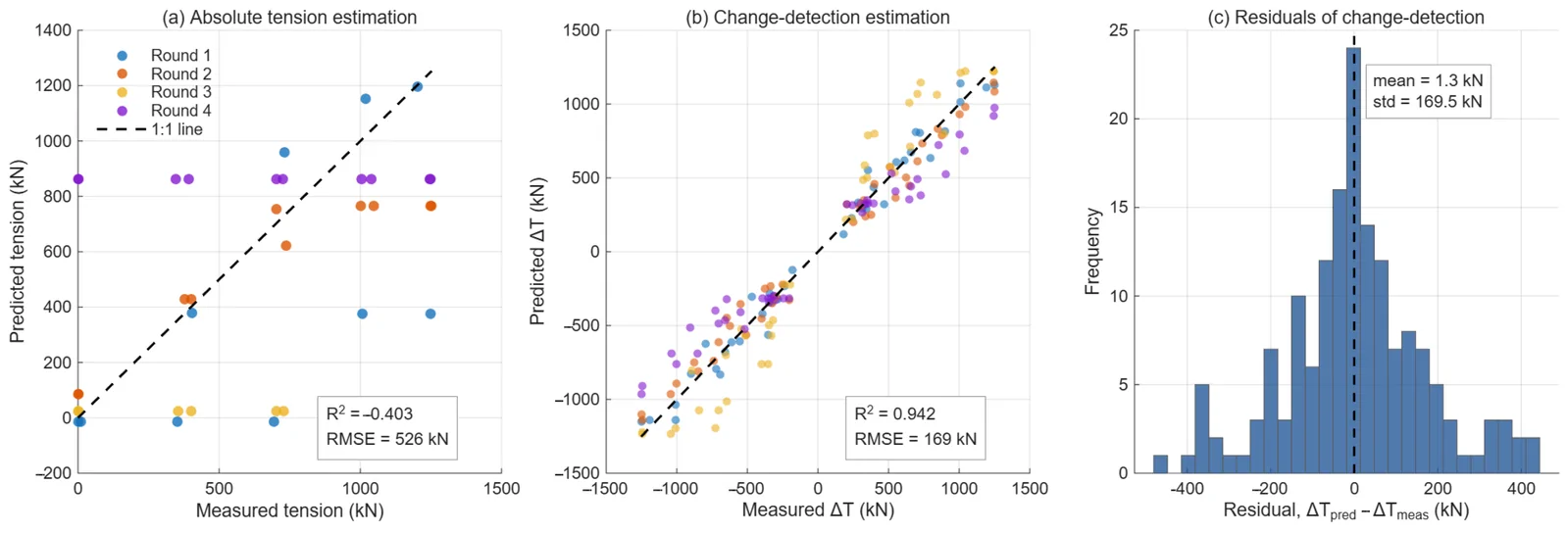
Absolute versus change-detection estimation for the same data and model (LSBoost; color denotes round): (**a**) absolute tension estimation, RMSE = 526 kN (42.0%FS); (**b**) change-detection estimation of tension change, RMSE = 169 kN (13.5%FS); (**c**) change-detection residual distribution. All axes are in kN. The predictions are those reported for LSBoost in Table 6.

Figure 6a shows this failure directly. Predictions cluster by measurement session rather than by actual tension, and within each session the prediction remains nearly constant even as the actual tension varies from zero to the maximum load. The model has learned the difference in signal level between sessions rather than the change caused by load. Because the same behavior appeared in GPR and LSBoost as well as in Ridge and PLS, the binding constraint lies in the representation of the input signal rather than in the expressive power of the regressor.

**Table 6:** Estimation performance under leave-one-round-out cross-validation: absolute versus change-detection representation.

Model	Absolute		Change Detection		
	*R* ^2^	RMSE (%FS)	*R* ^2^	RMSE (%FS)	1.96 × RMSE (kN)
Ridge	<0	794.1	0.884	19.1	468.1
PLS	<0	736.3	0.881	19.4	475.2
GPR	<0	53.2	0.736	28.8	705.9
LSBoost	<0	42.0	0.942	13.5	331.2

Full scale is 1252 kN (127.7 tf). The last column is 1.96 RMSE, a nominal error scale under distributional assumptions that this dataset does not establish; it is not a validated minimum detectable change. For LSBoost under the change-detection representation the RMSE is 169.0 kN (17.23 tf).

### 4.4. Within-Session Tension-Change Estimation

To remove the between-session variation of the absolute signal, the estimation target was redefined from absolute tension to the tension change relative to a reference state. For two measurement states *i* and *j* within one session, a regression model was constructed with the multichannel Hall voltage difference $\Delta V=V_{i}-V_{j}$ as input and the tension difference $\Delta T=T_{i}-T_{j}$ as output. Taking the difference within a session cancels the offset of that session and therefore eliminates variation caused by differences in initial magnetization state. Loading and unloading conditions were treated as separate sessions to prevent contamination by hysteresis. Pairing the five states within each of the eight sessions gives 80 unordered pairs, represented in both directions as 160 ordered differences. The reversed member of a pair is the negative of the other and carries no additional information, and the unordered pairs themselves share underlying states, so neither figure is a count of independent measurements; the experimental basis remains 40 acquired states from four rounds on one specimen.

Table 6 compares the two representations under leave-one-round-out cross-validation. With absolute signals all models returned $R^{2}<0$ and RMSE from 42.0%FS to 794.1%FS. With differential signals every model recovered a valid estimation performance, and LSBoost was best at $R^{2}=0.942$ and RMSE =169.0 kN (17.23 tf), or 13.5%FS, for which 1.96RMSE is 331.2 kN. The improvement is consistent with cancellation of the session offset by the difference operation. It was obtained without the load labels of the held-out round, which tests prediction on a round not used for fitting; the channel screening and the regression were nonetheless developed from known-load measurements on this specimen, so the result does not show that the procedure works without calibration.

One caution applies to reading these two columns against each other. The coefficient of determination is referred to the variance of whichever target is being predicted, and the signed tension change has a wider spread than the absolute tension from which it is formed; for five load steps spread evenly over the range, the mean square of the signed differences is 2.5 times the variance of the absolute levels. The same absolute error therefore yields a higher $R^{2}$ against ΔT than against *T*, and the two $R^{2}$ columns are not interchangeable measures of physical resolution. The comparison that does carry weight is between the RMSE values, which share the full-scale denominator.

The spread between models under the differential representation, from $R^{2}=0.736$ to 0.942, is far smaller than the change produced by switching representation, for which the same models moved from complete failure to valid estimation. Securing an appropriate signal representation is therefore the precondition, and model selection is a secondary optimization performed on top of it.

Under the present conditions LSBoost was best on all three metrics, so it is the appropriate choice when the training data span the tension range of the target and multiple sessions are available. LSBoost cannot extrapolate beyond the training range by construction, however, so where the target tension may exceed that range or where the number of sessions is limited, Ridge is more suitable: it performs close to the best model while allowing physical interpretation of its coefficients and prediction of its extrapolation behavior. Model selection is therefore governed by application conditions rather than by a single optimum.

Figure 6 contrasts the two representations for the same data and the same model. Under the differential representation, predictions from all sessions distribute about the y=x line and the session clustering seen in Figure 6a is resolved, which visually confirms removal of the session-specific offset. The residual distribution in Figure 6c is symmetric about zero with no systematic bias. Vertical scatter remains at each level of tension change, arising from session-to-session gain variation that the difference operation does not remove; the between-session coefficient of variation of the fitted slope ranged from 11.9% to 34.9%, with a median of 23.3%. No inference is drawn from comparing that figure with the 13.5%FS error, because a coefficient of variation is normalized by the slope and the error by full scale. The contribution can be measured directly instead. Fitting a gain and an offset to each held-out round and removing them lowers the RMSE from 169.0 kN to 119.8 kN, that is from 13.5 to 9.6%FS, so round-to-round variation in sensitivity accounts for 49.7% of the residual variance. This is an oracle decomposition: the gain is fitted on the labels of the held-out round, so 119.8 kN is not an accuracy that the method achieves. It bounds what any session-level gain calibration could recover, and it identifies gain variation rather than noise or thermal drift as the largest single component of the error.

**Figure 7:**
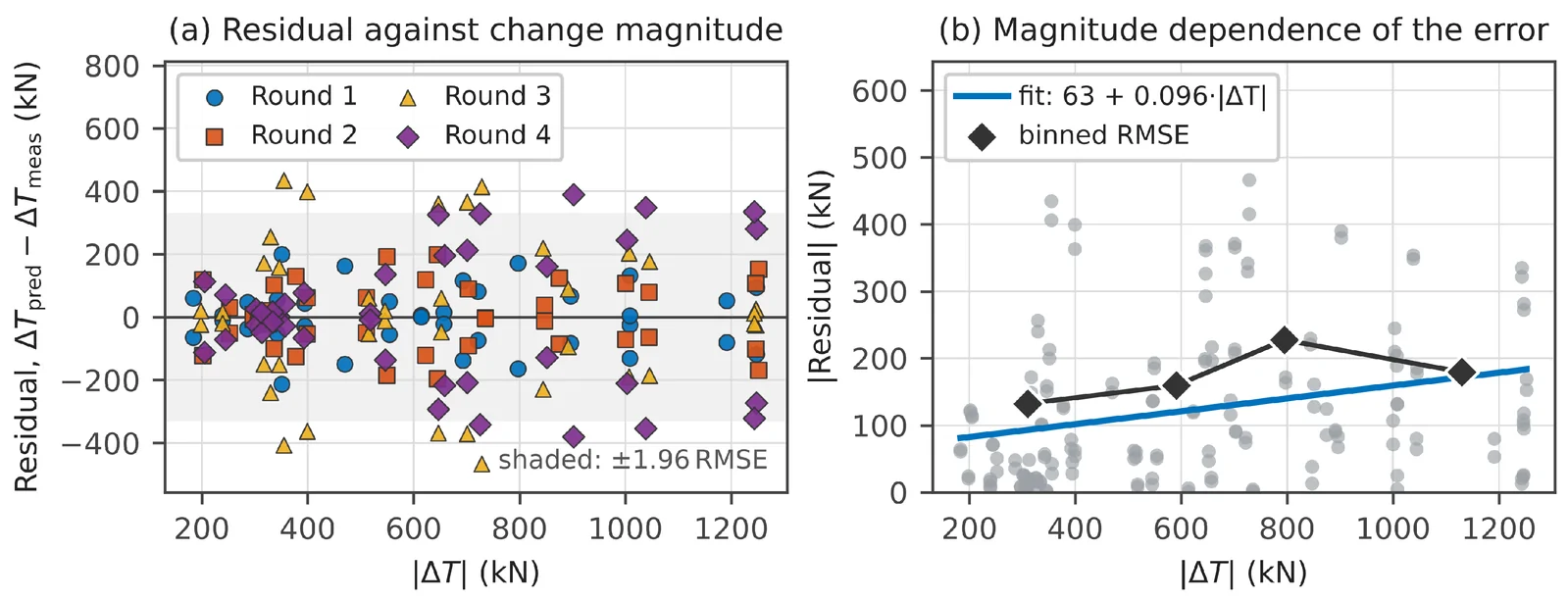
Dependence of the estimation error on the magnitude of the tension change, for the change-detection model under leave-one-round-out cross-validation: (**a**) signed residual against |ΔT|, by held-out round, with the band ±1.96 RMSE shaded; (**b**) absolute residual against |ΔT|, with the individual differential samples in grey and, computed over them, a least-squares line and the root-mean-square error within four bins of |ΔT|. Colours identify the rounds as in Figure 6, and marker shapes repeat that identification for monochrome reproduction.

A multiplicative error of this kind should grow with the size of the change it acts on, and Figure 7 tests that directly.

The error does grow with the change, and by about the amount a gain error predicts. The absolute residual correlates with |ΔT| at +0.27, and a least-squares fit gives 63+0.096|ΔT| in kilonewtons: a floor of about 63 kN independent of the change, plus a term equal to 9.6% of the change itself. Binned by magnitude, the root-mean-square error rises from 132 kN below 400 kN of change to 227 kN between 700 and 1000 kN. The proportional term is the signature of a session gain differing from the one learned, and its size is consistent with the fitted slopes of Table 7.

Two qualifications follow from the same figure. The relationship is not uniform across rounds: it is strongest in round 4, whose fitted slope of 0.74 departs furthest from unity and whose within-round correlation reaches +0.80, and it is absent in round 3, where the largest residuals occur at small changes instead. A single proportional model therefore does not describe every round. The intercept of 63 kN is also not removed by any gain correction, and it is of the same order as the floor inferred from the channel-count sweep in Section 4.2; both point to a component common to the whole array at a given installation.

### 4.5. Performance of the Individual Folds

The pooled figures conceal a structure that bears on how they should be read. Table 7 reports each held-out round separately.

The bias reported over both directions of each pair is not evidence that the error is unbiased. Each unordered pair enters the evaluation as (i,j) and as (j,i), the residuals of the two directions correlate at −0.996, and their mean therefore cancels by construction. Recomputed over the direction in which tension increases, the bias is −1.9, −55.0, +158.0 and −139.5 kN for rounds 1 to 4, and −25.4 kN for the loading sessions against +6.2 kN for the unloading sessions. The error does contain a systematic component, and the claim that it does not has been withdrawn.

The dispersion differs by more than a factor of two between folds: rounds 1 and 2 give 92.2 and 102.3 kN, rounds 3 and 4 give 227.5 and 208.5 kN. That division is the division of the design. Rounds 1 and 2 were acquired at zero eccentricity and rounds 3 and 4 at −50 and +50 mm, so every fold holding out a zero-eccentricity round retains a round at the same position in training, whereas the folds holding out rounds 3 and 4 do not. The fitted slopes, 0.99, 0.90, 1.18 and 0.74, and the one-directional biases follow the same ordering: a held-out round whose sensitivity exceeds the value learned from the remaining rounds is over-predicted, which is round 3, while the training set of fold 4 contains that high-sensitivity round and round 4 is correspondingly under-predicted.

Evaluating the two conditions separately gives Table 8. Restricting both training and testing to the two zero-eccentricity rounds gives 110.6 kN, that is 8.8%FS. Training on those rounds and predicting a displaced round gives 273 kN at −50 mm and 141 kN at +50 mm. The pooled value of 169.0 kN is the quadratic mean of two different conditions and is not the accuracy obtainable at a fixed installation position.

At zero eccentricity the regularized linear model reaches 110.7 kN against 110.6 kN for the boosted trees. The advantage of the nonlinear model is therefore specific to the pooled set, in which the displaced rounds are present, and it supports rather than contradicts the conclusion of Section 4.4 that securing the signal representation precedes the choice of model.

The same split answers a question the differencing scheme leaves open: what happens when the reference state is not taken from the same session. Three cases can be distinguished, and they degrade in order. The protocol is the one of Section 4.4 throughout: the model is trained on the within-session pairs of the two zero-eccentricity rounds, and only the source of the test pairs changes; loading and unloading are kept separate so that the pairing introduces no hysteresis. Within a session, which is the case reported above, the estimate is 110.6 kN at zero eccentricity. Taking one state of each pair from one round and the other from the other, so that the reference comes from a separate acquisition at the same position, degrades it to 627.0 kN, or 50.0%FS, with $R^{2}$ of −0.009. Drawing the two states from rounds acquired at different eccentricities fails further still, giving 1091 and 1231 kN, that is 87.1 and 98.3%FS, with $R^{2}$ of −2.05 and −2.88.

One subset of those cross-round pairs carries the practical consequence and is therefore reported on its own. Fourteen of the hundred pairs join two states whose tension differs by less than 2 tf, 0.57 tf on average, which is 5.6 kN: the load is to that precision unchanged, and the two states differ only in being separate acquisitions. For these the model reports changes with a root-mean-square of 748.7 kN and a largest value of 990.0 kN. A tension that did not move is thus reported as having moved by a substantial fraction of full scale, and the figure is not an error about the size of a change but an indication of change where there was none. This is the quantity that governs whether the method could be used to compare measurement epochs, and on the present evidence it could not.

The mechanism follows the same order. Within a session the signal offset is common to the two states and cancels identically. Between two acquisitions at the same position it does not: for the selected channels the between-round spread of the offset is a median 7.1 times the tension-induced swing within a session, and 6.9 times with the one channel whose offset spread is an outlier set aside. Between two acquisitions at different positions the sensitivity differs as well, by 26.2% at −50 mm, and no differencing scheme removes that term. Removing each round’s offset using the held-out labels, an oracle operation that bounds what any offset calibration could achieve, recovers $R^{2}=0.360$ and 499.3 kN, which is still 39.9%FS. What remains is the gain. The method as demonstrated is therefore a within-session change detector, and extending it to compare measurement epochs requires calibration of the sensitivity and not of the offset alone.

### 4.6. Ablation of the Temperature Covariates

Section 3.5 introduced seven temperature covariates on the grounds that the channels differ in thermal sensitivity. Whether they improve the estimate is a separate question, and withholding them answers it. Table 9 reports the same cross-validation under four input sets.

The covariates did not contribute. Withholding them left the boosted-tree model unchanged, from $R^{2}=0.942$ to 0.943, and improved the regularized linear model from 0.884 to 0.931, that is from 19.1 to 14.7%FS. The negative control, in which the Hall signals are withheld and only the temperatures are supplied, returns $R^{2}$ near zero for both models.

That control removes an input and therefore does not test whether recomputing the channel-quality index inside each training fold leaks label information. A permutation test does. The tension labels were permuted within each session, which preserves the experimental design and the distribution of tension differences and destroys only the association between signal and load, and the entire procedure—pair construction, channel selection, standardization, hyperparameter selection and prediction—was repeated on each permuted dataset. Over 300 permutations the null distribution of $R^{2}$ has mean −0.16, a 95th percentile of +0.06 and a maximum of +0.25, against an observed $R^{2}$ of +0.93; the empirical *p*-value is 0.003, the lower bound attainable with 300 permutations. No label information reaches the models through the selection step.

The reason is the size of the thermal excursion rather than the sensitivity. Within a session the temperature never moved by more than ±0.6 °C, because current is applied only while a load step is acquired. Differenced over such a span the covariates carry almost no information while adding seven degrees of freedom; the regularized linear model pays that cost directly, whereas the boosted trees discard the uninformative splits. The covariates are retained in the reported configuration as a provision for field conditions, where the excursion would be far larger, but this dataset establishes neither their benefit nor their cost under a realistic diurnal cycle.

One consequence bears on model selection. On a common input set the margin of LSBoost over Ridge narrows from 5.6 to 1.4%FS. LSBoost remains the better performer and is the model used for the figures, but the case for a nonlinear estimator is weaker than Table 6 alone suggests: a regularized linear model operating on the differenced Hall signals reaches within 1.4%FS of it, with coefficients that can be inspected and extrapolation behavior that can be predicted.

A thermal contribution acts on the differential signal as well. Its share cannot be separated from the gain share by this experiment, because temperature and load were not varied independently: the core heats while the load is raised, so the two are confounded in the data from which the estimate is formed. What can be said is bounded. The intra-session excursion never exceeded ±0.6 °C, and at the largest per-channel sensitivity of Figure 4b, 0.54 mV/°C, that corresponds to at most 0.32 mV against a tension-induced change of about 2.3 mV. That is 14% of the signal and does not exclude a thermal contribution; it bounds it. The ablation of Section 4.6 is consistent with this: removing the temperature covariates does not degrade the estimate. Neither result establishes that the covariates remove a thermal effect, only that no benefit from them is detectable over this excursion, and Section 4.9 records the separation as unresolved.

A second term acts through the supply. The DRV5055 output is ratiometric over its full span, so the standing output of about 2747 mV scales with the 5 V rail and a change of only 0.084% in that rail would equal the entire tension-induced change of 2.3 mV. The rail is supplied by the acquisition module and was not recorded, so it cannot be subtracted. It can, however, be bounded from the measurements themselves. Regressing each channel on load within a session and extracting the component of the residual common to all channels in proportion to their standing output—the signature a supply change would produce—gives a within-session variation of 34 ppm, that is 0.0034%, corresponding to 0.094 mV or 4.1% of the tension-induced change. The supply was therefore stable enough over the few seconds of an acquisition for this term not to dominate, although it cannot be excluded at the few-per-cent level.

Between rounds the supply requires a more careful statement than within a session. The sensitivity of a ratiometric element is proportional to its supply, so a rail that differs between two acquisitions scales the gain of the array in the same proportion, and the tolerance of the rail quoted in Section 3.3 is about ±4%, which is not small beside the between-round variation of the fitted slope. A supply difference may therefore contribute to that variation, and with the rail unrecorded this work cannot determine how much. What a supply difference cannot produce is the structure of the variation. Acting in the same proportion on every element, it leaves the ratio of one channel’s sensitivity to another’s unchanged, whereas that ratio varies between rounds by 13 to 16% from channel to channel, and Section 4.5 attributes that part to the change of array eccentricity. The division is therefore between a common factor, which a supply difference could supply and which the present data cannot separate from a thermal or magnetic one, and a channel-dependent pattern, which it could not. Section 4.9 records that the supply should be logged in future measurements, which would resolve the first of the two.

The sensitivity temperature coefficient of the element can be tested against the same variation, and it does not survive the test. Between the sessions acquired at zero eccentricity, which are the ones that isolate reproducibility at a fixed position, the mean temperature of the array spans 2.17 °C. At 0.12%/°C that span predicts a sensitivity change of 0.26%, against an observed between-session variation of the fitted slope of 8.8%. The coefficient therefore accounts for about 3% of what is observed, and reproducing the observed figure from temperature alone would require a between-round difference of about 73 °C, which the measurement does not contain. Of the two instrument-side mechanisms raised here, the ratiometric supply is the one the present data cannot bound and the temperature coefficient is the one they can.

**Figure 8:**
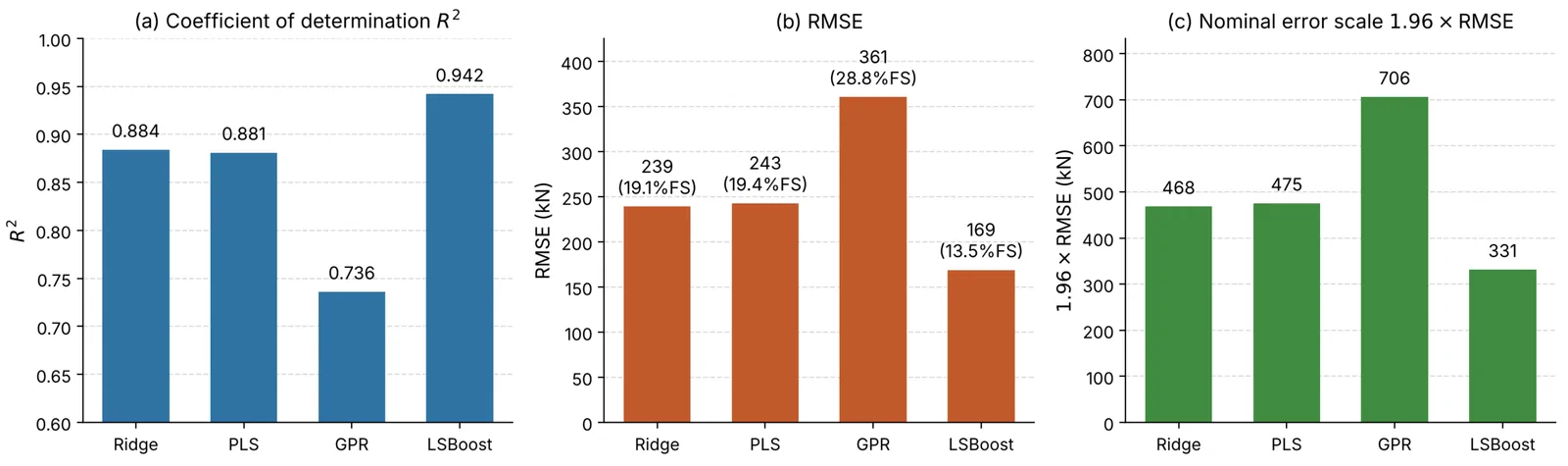
Change-detection performance by model: (**a**) $R^{2}$; (**b**) RMSE in kN, with the percentage of full scale annotated; (**c**) the nominal error scale 1.96×RMSE in kN. The quantity in panel (**c**) is a scaled cross-validated error, not a validated minimum detectable change, as Section 4.8 states. For the best model the RMSE is 169.0 kN (13.5%FS).

Figure 8 compares the coefficient of determination, the RMSE in percent of full scale and the scaled error across the four models. The three panels rank the models identically, which is expected because the third quantity is a fixed multiple of the RMSE and the full-scale normalization is common to all models; the panels are retained because the three are read differently in practice, $R^{2}$ against the spread of the reference, RMSE against the measurement, and the scaled value against the magnitude of a change that would have to be distinguished. The separation between LSBoost and the two linear models, 13.5%FS against 19.1 and 19.4%FS, is smaller than the separation between either of them and GPR at 28.8%FS, and all four lie within a factor of about two of one another, in contrast with the two orders of magnitude that separate the absolute and differential representations of the same models.

### 4.7. Comparison with Existing Tension-Measurement Methods

The accuracy reported above is best read against what a method requires before it can be applied at all, because the published methods differ more in their applicability than in their error. Table 10 sets the present result beside a recent resonance-enhanced magnetoelastic system, which reports errors of 5.97% on prestress loss and 13.26% during construction. That arrangement, like through-type elasto-magnetic sensing generally, requires a sensor to be installed on or around the strand before or during construction, and requires a calibration against known load. A conventional embedded through-type arrangement [21] likewise uses known-load laboratory training and sensors fitted around the tendon sheath before casting, and has been applied on a full-scale girder; it is included in Table 10 for the installation and training conditions it requires rather than for a numerical comparison. The present system reports 13.5%FS, which is of the same order, and does not require a sensor to be installed before casting. It does, however, require known-load training data: the channel screening and the regression are both developed from measurements of known tension on the same specimen, and what the cross-validation withholds is the labels of one tensioning round, not the calibration itself. The distinction that the present work supports is therefore one of installation, not of calibration. Whether the procedure transfers to a member of unknown tension and a different installation has not been demonstrated.

A further qualification applies. The reference quantity in this work is the total force of one duct measured at the anchorage, so the resolution reported is a resolution on that total, as Section 4.8 notes, whereas through-type sensors report the force of the individual strand they encircle.

### 4.8. Nominal Error Scale and Detection Limits

For maintenance, the governing metric is the smallest tension change that can be detected reliably, and the present data bound it only loosely. The cross-validated RMSE of the best model is 169.0 kN, or 13.5% of the 1252 kN full-scale reference; multiplying by 1.96 gives 331.2 kN, or 26.5%FS. That product assumes errors that are unbiased, normally distributed and of constant variance, and the residuals satisfy only the first of the three. The interval ±1.96 RMSE contains 90.0% of the residuals rather than 95%, and the empirical 95th percentile of the absolute residuals is 367.8 kN, or 29.4%FS. The normal-theory figure is therefore optimistic by about 11%, and both are reported here.

The cause is not asymmetry. The residual distribution is very nearly symmetric, with a skewness of +0.005, but it is leptokurtic, with an excess kurtosis of +0.49. That is what pooling folds of unequal dispersion produces: as Table 7 shows, the residual standard deviation ranges from about 93 to 231 kN between folds, and a mixture of normal distributions with different widths has heavier tails than any one of them. The quantity is reported as an error scale rather than as an experimentally verified minimum detectable loss; false-alarm and detection probabilities near it have not been measured. The reference quantity throughout is the total force of the twelve-strand tendon in one duct, measured at the anchorage by the load cell, so the figure applies to that total force and not to individual strands.

Expressed against the tendon itself the figures read as follows. The twelve strands share the 1252 kN full-scale force, so one strand carries 104 kN, and the pooled error scale of 331 kN is equivalent to the loss of 3.2 strands. At a fixed installation position, the condition isolated in Table 8, the error scale falls to 217 kN, equivalent to 2.1 strands. A loss of two strands in twelve is already advanced section damage rather than an early indication, so the sensitivity demonstrated here addresses deterioration that has progressed, not its onset. Two qualifications attach to the comparison. It assumes that the intact strands do not take up the force released by a failed one, which in a bonded tendon they partly do over a development length, so the force actually missing at the measured section is smaller than the force lost at the break. And the quantity compared is a scaled cross-validated error, not a detection limit established against controlled changes of that size.

Detection accuracy computed against a threshold of 5%FS (±62.6 kN) was 98% to 100%, but this figure requires care. The load steps in this test were discrete and the tension change of every differential sample exceeded the threshold by a wide margin, so no case near the threshold was present in the data. Detection capability near a decision threshold is therefore not verified by this figure, and it is not used as evidence of small-loss sensitivity. Establishing that capability requires controlled tension changes of a few per cent of full scale, which the present loading protocol did not produce.

It is sometimes argued that repeated measurement relaxes such a limit, because random error averages out over a trend. That argument does not carry here for two reasons. The error analysed above is not predominantly random: a session-dependent gain acts systematically within a session and changes between sessions, so averaging within one installation does not reduce it and averaging across installations is exactly the case not tested. More fundamentally, every difference evaluated in this work was formed between two states of the same loading session, acquired minutes apart under one installation of the magnetizer. Comparing two measurement epochs separated by months, with the device removed and refitted in between, is a different problem: it requires the offset and the gain to be preserved across that interval, which the between-session variation reported in Section 4 shows they are not. Section 4.5 quantifies it: drawing the reference from a separate acquisition at the same position raises the error from 110.6 to 627.0 kN, and drawing it from a different position fails outright. Nominating the first field measurement as a relative reference does not by itself preserve them. Long-term trend detection is therefore outside what the present evidence supports.

### 4.9. Limitations

The experimental scope is the principal limitation and is stated first. All measurements come from one 2 m specimen at one embedment depth, 80 mm, with one stirrup spacing, 300 mm, one duct and one strand configuration of twelve 12.7 mm strands. Depth and cover, strand diameter and the number of strands per duct were therefore not varied experimentally, and the tendon lengths and the range of cover thicknesses found in service bridges are not spanned. The specimen itself contains embedment depths of 150 and 300 mm and a stirrup spacing of 150 mm in other regions, so these variables are accessible without new fabrication, and measuring them is the immediate extension of this work. The finite-element model varied the standoff only between 80 and 100 mm, which is narrower than the range of practical interest.

The treatment of ordinary reinforcement is a second limitation of the same kind. The finite-element model of Section 2.5 places the magnetizer, the duct and the strand explicitly but assumes that the surrounding reinforcement does not alter the flux reaching the duct, and the experiment reduced that influence by centering the array within a 300 mm stirrup spacing rather than by quantifying it. Multi-layer reinforcement meshes, stirrups at closer spacing and non-prestressed bars near the measurement surface will distort the field and may shift the spatial distribution of the channel-quality index. Their effect has been characterized for measurements that target the reinforcement itself [12,13] but not for measurement of a tendon beneath it. A controlled comparison across the 300 and 150 mm stirrup regions of the present specimen, together with a finite-element study in which the reinforcement is modelled explicitly, is required before the method can be transferred to an arbitrary section.

Four tensioning cycles and 40 acquisitions are a small sample, and although the differential formulation yields 160 ordered pairs, those pairs are not independent; the cross-validated figures should be read as estimates from four independent folds.

The lateral position of the array is a measured variable in this work rather than an untested one, but it was measured at three values only. Rounds 1 and 2 were acquired with the array centered on the tendon and rounds 3 and 4 with it displaced −50 and +50 mm perpendicular to the tendon, and Section 4.5 reports the two conditions separately: 110.6 kN when training and testing share the centered position, against 273 and 141 kN when a model trained at the centered position is applied to the displaced rounds. The cost of a 50 mm lateral error is therefore bounded, but three things are not. The behavior between 0 and 50 mm was not sampled, and nothing in these data establishes that it is monotonic; offsets larger than 50 mm were not applied; and the two remaining degrees of freedom of the installation, displacement along the tendon and rotation of the array about the magnetizer axis, were held nominally fixed and were not varied at all. The asymmetry between the two directions is itself unexplained: the selected channels are 26.2% more sensitive at −50 mm and 1.9% more sensitive at +50 mm than at the centered position, which is consistent with the tendon not lying on the nominal axis of the duct, but the position of the tendon within the duct was not measured and the explanation is not established.

The supply voltage of the Hall array was not recorded, and it should be in any further measurement. The DRV5055 output is ratiometric to its supply, so a change in the 5 V rail scales every channel in the same proportion and is indistinguishable from a common-mode signal. Section 4.6 bounds that term from the acquired data rather than measuring it, limiting a supply excursion to 34 ppm, or 4.1% of the tension-induced change, from the common-mode drift of the standing outputs. Logging the rail on a spare analog input, which costs one channel, would replace the bound with a correction and would also remove the ambiguity between a supply change and a genuine common-mode field change.

The temperature covariates could not be evaluated on this dataset. The intra-session excursion never exceeded ±0.6 °C, and withholding the covariates changed the boosted-tree accuracy by 0.2%FS while improving the linear model by 4.4%FS, as Section 4.6 reports. They are retained as a provision for field conditions, but neither their benefit nor their cost under a realistic diurnal cycle can be established here, and temperature and load were not varied independently, so the thermal share of the residual remains unseparated from the gain share.

The residual is dominated by variation in sensitivity between rounds rather than by measurement noise. Each reported value is the mean of about $3.8\times 10^{5}$ samples, which places electronic noise below one microvolt against a tension-induced change of about 2.3 mV, so instrumentation noise is not the limiting term. Removing a per-round gain and offset lowers the RMSE from 169.0 to 119.8 kN, so round-to-round sensitivity variation accounts for 49.7% of the residual variance; this is an oracle decomposition fitted on held-out labels and is not an achievable accuracy. Consistently with it, adding channels does not remove the term, the RMSE falling as $K^{-0.16}$ rather than $K^{-1/2}$.

The reference quantity itself carries a limitation that the present arrangement cannot resolve, and the ungrouted duct makes it specific. The load cell measures the total force at the anchorage, whereas the magnetized region is a short length of tendon away from it. In the ungrouted specimen used here the two are separated only by the tendon path and by friction against the duct wall over a 2 m straight length, which keeps the discrepancy small, but the relationship was not measured. A grouted in-service tendon is a different case: bond transfers force to the surrounding grout continuously along the member, so the anchorage force need not represent the local force in the magnetized region at all, and a change measured locally need not correspond to a change at the anchorage. Because the duct was deliberately left ungrouted to permit repeated loading, this work provides no evidence on that transfer, and it is the condition that a field application would actually meet.

Three further limits bear on how the result should be read. First, the procedure is not calibration-free. Both the channel screening and the regression are developed from measurements of known tension on this specimen, and the cross-validation withholds the labels of one round rather than the calibration itself; transfer to a member of unknown tension has not been demonstrated. Second, every difference evaluated here was formed between two states of the same session, acquired minutes apart under one installation, so nothing in this work tests whether an offset or a gain is preserved across measurement epochs or across a removal and refitting of the device, which is the condition a monitoring application would face. Third, the magnetomechanical model is used as a qualitative interpretation: its material law was identified on a single strand in a through-type coil, its transfer to the twelve-strand duct assembly is assumed, the predicted local fields were not measured, and no mesh-convergence documentation is reported.

The difference operation cancels the session offset but not the variation of gain, the sensitivity to tension; the between-session coefficient of variation of the fitted slope had a median of 23.3%, so error dependent on session and installation condition remains in the magnitude estimate. The discrete load steps of the laboratory test left detection capability for small changes substantively unverified. The temperature characteristics were obtained under self-heating of the magnetizer, and validity under seasonal ambient conditions was not verified. Validation was confined to laboratory conditions in which a load cell provides the reference tension; field verification on a real bridge was not performed.

## 5. Conclusions

An external-magnetization elasto-magnetic measurement system was developed to evaluate the tension of internal tendons in aged PSC bridges on which no sensor was pre-installed, and its performance was examined through laboratory tensioning tests. The following conclusions are drawn.

### 5.1. What the Measurements Establish

A triple concentric ferrite solenoid array of radii 50, 100 and 150 mm and a 25-channel Hall and thermal measurement unit were built and operated on one 2 m specimen at the nominal 80 mm embedment with 300 mm stirrup spacing. The reference was the total anchorage force of one twelve-strand tendon, and four loading–unloading rounds yielded 40 acquired states.

Estimating tension directly from absolute Hall voltages failed to generalize across rounds, the error exceeding the spread of the reference itself. The failure was common to all four models, from Ridge and PLS through GPR to LSBoost, which locates the problem in the signal representation rather than in the choice of regressor. Forming differences between two states of the same session recovered prediction on a withheld round, giving $R^{2}=0.942$ and RMSE =169.0 kN, that is 13.5% of the 1252 kN full scale. This is the principal experimental result and it is specific to the tested specimen and acquisition protocol.

Channel effectiveness was found to depend on angular position as well as radius, and the eight channels retained in each fold were distributed across all four radial groups, so a fixed geometric grouping would not have identified them. The screening criterion that produced this ranking is supervised, being computed from reference tension changes.

Three further results follow from the same dataset. The temperature covariates did not improve the estimate: withholding them left the boosted-tree model unchanged and improved the linear model by 4.4%FS, the intra-session thermal excursion being at most ±0.6 °C. Increasing the number of retained channels lowers the error as $K^{-0.16}$ rather than as $K^{-1/2}$, which places a floor of about 128 to 157 kN that is common to the whole array and cannot be averaged away. Removing a fitted per-round gain and offset lowers the RMSE from 169.0 to 119.8 kN, so variation in sensitivity between rounds accounts for about half of the residual variance.

In the calculated axial field, the nested arrangement delivered about 3.2 times the on-axis flux density of three coplanar coils carrying the same total ampere-turns. Against a single coil of the same outer diameter the ratio is accounted for by the additional ampere-turns that nesting permits within the envelope, not by the arrangement.

### 5.2. What the Model Suggests and What Remains Unverified

The coupled magnetomechanical model, whose strand material was calibrated against through-type measurements on a single strand, offers a qualitative reading of how a field applied normal to the member surface couples to a strand lying perpendicular to the magnetizer axis: the computed component along the strand is antisymmetric about the mid-plane of the array and varies with tension, falling inside the footprint of the windings and growing outside it as the permeability of the strand is reduced, and the small longitudinal demagnetizing factor of a slender strand favours a longitudinal internal field. Transfer of the single-strand material law to the twelve-strand bundle inside a steel duct is assumed rather than demonstrated, the predicted local fields were not measured, and the surrounding reinforcement was not represented.

The product 1.96×RMSE, 331.2 kN or 26.5%FS, rests on distributional assumptions the residuals do not satisfy: the interval contains 90.0% of them rather than 95%, and the empirical 95th percentile is 367.8 kN. It is not a verified minimum detectable loss. Detection near a decision threshold, transfer across specimens, embedment depths and reinforcement layouts, and preservation of a reference between measurement epochs all remain unverified, and the differences evaluated here were formed within single sessions minutes apart under one installation.

Reducing gain variation is the first priority for accuracy, and the decomposition above makes the target quantitative. Because round-to-round gain variation accounts for roughly half of the residual variance and is not reduced by adding channels, the effective improvement is not a larger array or a different estimator but a means of determining the sensitivity of each installation independently. Three candidates follow from the likely origins of the common-mode term: monitoring the excitation current directly rather than relying on the compensation of the supply; measuring the standoff between the array and the tendon at each installation; and placing a reference element of known magnetic response within the array against which the session gain can be normalized. A method is also under review in which the residual-magnetization signal measured with no magnetizing current applied is used to estimate the gain of that session, which requires no load information. Were such a calibration to recover the gain term in full, the error would fall from 13.5 to about 9.6%FS and the nominal error scale from 331.2 to about 235 kN. The zone temperature to Hall voltage relationships obtained here will be extended to the seasonal ambient range, and a procedure established for correcting the temperature difference between the reference and re-measurement epochs in long-term monitoring. A parametric extension of the finite-element model is planned over embedment depths from 50 to 300 mm, strand diameters of 12.7 and 15.2 mm, and duct populations of 7, 12 and 19 strands, with multi-layer reinforcement and stirrups modelled explicitly, so that the admissible cover range can be stated as a signal-to-noise criterion rather than as the single depth examined here. The specimen also contains regions at 150 and 300 mm embedment and at 150 mm stirrup spacing, which would allow those conditions to be measured without new fabrication; no results from them are reported here. Loading tests on a full-scale 25 m PSC I-girder are intended to examine the effect of member dimensions and reinforcement layout, and repeated measurement over time is required before any claim about trend-based detection can be made. Finally, field applicability will be verified on an in-service PSC bridge, evaluating the model with temperature covariates where a seasonal difference exists between epochs, and examining operational procedures for establishing a reference state either by simulation of the zero-stress signal or by adopting the initial measurement epoch as the reference.

## Figures and Tables

**Table 1:** Configuration and analysis of the earlier external-magnetization study and of the present work.

Aspect	Previous Study [24]	Present Investigation
Magnetizer	Eight coils placed on both sides of the girder	Three concentric ferrite-core coils on one accessible surface
Sensing unit	Four InSb Hall elements (HW302B, AKM), battery powered at 1.5 V	25-channel printed board of DRV5055A4-family elements at 5 V
Specimen	6.6 m scaled I-girder, three tendons evaluated	2 m specimen, one target tendon evaluated
Excitation	10 Hz square wave of 1.5 V amplitude from a function generator through a power amplifier	10 A direct current per coil
Numerical model	Magnetization of reinforcement, duct and tendon in ANSYS Maxwell 2021 R1	Coupled magnetic and mechanical analysis with a stress-dependent material law
Estimation	Normalization of a temperature-compensated tension index with quadratic regression	Supervised channel screening, and absolute voltages compared against within-session differences under cross-validation

**Table 2:** Specifications of the fabricated concentric solenoid magnetizer.

Coil	Outer Diameter	Turns	Measured Resistance	Dissipation at 10 A
DRH-100	100 mm	211.5	0.33826 Ω	33.8 W
DRH-200	200 mm	211.5	0.70490 Ω	70.5 W
DRH-300	300 mm	206.5	1.07015 Ω	107.0 W

**Table 3:** Coupled magnetomechanical finite-element model of the specimen.

Item	Setting
Software	COMSOL Multiphysics v6.2
Modules	AC/DC (magnetic fields, electrical circuit); Solid Mechanics
Winding	Four layers over a 100 mm length at radii 50, 100 and 150 mm
Excitation	10 A direct current per coil
Strand	Jiles–Atherton, $M_{s}=6.9\times 10^{5}$ A/m, $\lambda_{s}=-1.4$ ppm
Duct	Galvanized low-carbon steel, 0.4 mm wall, σ=8.41 MS/m
Concrete	$\mu_{r}=1$
Mechanical load	0 to 1112.7 MPa applied to the single strand used for material identification, fixed at the opposite end
Material-identification model (2D axisymmetric)	
Mesh	Physics-controlled, free triangular and mapped; 8244 elements; minimum element size 0.275 mm
Boundary conditions	Magnetic fields: free space in air. Solid mechanics: fixed end and boundary load
Solver	Time dependent, 0 to 2 s in steps of 0.0005 s, relative tolerance 0.001
Coil material	Copper, driven through the electrical-circuit module to couple primary and secondary windings

The model reproduces the radii, the winding length and the excitation of the fabricated magnetizer rather than its conductor geometry; the physical coils use 2.0 mm PEW wire in six layers, as listed in Table 2. The stress range is that of the single-strand material-identification test and is not the stress of the specimen tendon: under an assumption of equal load sharing the maximum total force of 1252 kN corresponds to an average strand stress of about 1057 MPa for the nominal steel area, and individual strand stresses were not measured.

**Table 4:** Radial and angular layout of the Hall-sensor array.

Group	Radius	Angular Positions	Count
Center	0 mm	center	1
Inner	55 mm	45°, 135°, 225°, 315°	4
Middle	75 mm	0°, 25°, 65°, 115°, 155°, 180°, 205°, 245°, 295°, 335°	10
Outer	125 mm	0°, 25°, 65°, 115°, 155°, 180°, 205°, 245°, 295°, 335°	10

**Table 5:** Specification of the Hall and acquisition chain.

Item	Value
Hall element	DRV5055A4 family (full ordering suffix not verified)
Hall supply	5 V
Hall sensitivity	12.5 mV/mT (nominal, not independently calibrated)
Quiescent output	$V_{\mathrm{CC}}/2$ (zero field)
Linear output range	0.2 V to $V_{\mathrm{CC}}-0.2$ V
Measuring range	±169 mT
Output noise	1.5 mV peak to peak; 130 nT/$\sqrt{\mathrm{Hz}}$ input-referred
Bandwidth	20 kHz
Acquisition unit	NI USB-6453, 32 single-ended channels, 16 independent ADCs
ADC resolution	20 bit
Input range	±5 V (software selectable), DC coupled
Input impedance	>10 GΩ; CMRR > 100 dB
Absolute accuracy	692 μV on the ±5 V range
Sampling rate	100 kS/s per channel, all 32 channels
Acquisition window	3.5 to 5.2 s per measurement (median 3.8 s)
Temperature element	NTC thermistor, converted to degrees Celsius

**Table 7:** Held-out performance of each cross-validation fold under the change-detection representation.

Held-Out	Ecc.	*n*	*R* ^2^	RMSE	Bias (kN)	Fitted
Round	(mm)			(kN)	Both/One Dir.	Slope
1	0	40	0.982	92.2	−2.2/−1.9	0.99
2	0	40	0.979	102.3	+2.8/−55.0	0.90
3	−50	40	0.896	227.5	+2.0/+158.0	1.18
4	+50	40	0.913	208.5	+2.7/−139.5	0.74
Pooled	—	160	0.942	169.0	+1.3/−9.6	—

The slope is obtained by regressing predicted on measured tension change within the fold. The pooled RMSE is the quadratic mean of the four fold values. Each unordered pair enters the evaluation in both directions, so the bias computed over both directions cancels by construction; the second bias column uses only the direction in which tension increases. Separated by session type, that bias is −25.4 kN for the loading sessions and +6.2 kN for the unloading sessions.

**Table 8:** Change-detection performance separated by the lateral eccentricity of the array (LSBoost).

Condition	Train	Test	*n*	*R* ^2^	RMSE (kN)	1.96 RMSE (kN)
Fixed position	1 ↔ 2	the other	80	0.975	110.6	217
Extrapolation to −50 mm	1, 2	3	40	0.850	273	535
Extrapolation to +50 mm	1, 2	4	40	0.960	141	276

Full scale is 1252 kN, so 110.6 kN is 8.8%FS, 273 kN is 21.8%FS and 141 kN is 11.3%FS. The two offsets are not symmetric: the selected channels are 26.2% more sensitive at −50 mm than at zero eccentricity and 1.9% more sensitive at +50 mm. Taken over the zero-eccentricity rounds alone the coefficient of variation of the channel sensitivity is 8.8%, against 15.8% over all eight sessions, so the between-session figure quoted in Section 4.4 is not a measure of reproducibility at a fixed position. The magnetizer was not removed between rounds 1 and 2, as Section 3.4 states, so the fixed-position row covers reproducibility between acquisitions and not between installations.

**Table 9:** Effect of the temperature covariates, under the same leave-one-round-out protocol.

Input Set	Ridge		LSBoost	
	*R* ^2^	RMSE (%FS)	*R* ^2^	RMSE (%FS)
ΔV only	0.931	14.7	0.943	13.3
ΔV + six zone temperatures	0.885	19.0	0.943	13.4
ΔV + all seven temperatures	0.884	19.1	0.942	13.5
Temperatures only	−0.046	57.3	−0.108	59.0

**Table 10:** Reported performance of magnetic tension-measurement methods for prestressing steel.

Method	Sensor Placement	Prior Calibration	Reported Error	Error Definition
Resonance-enhanced magnetoelastic, construction and prestress-loss stages [15]	Embedded on the tendon	Required	13.26%/5.97%	Of monitored prestress, precise calibration method
Embedded through-type EM with machine learning [21]	Embedded around the tendon sheath before casting	Known-load laboratory training	Not compared here	Reported on a different basis; see note
External magnetization, this work	Surface mounted, after construction	Known-load training required; target-member transfer unverified	13.5%	Of full scale, leave-one-round-out cross-validation

The table compares installation and training conditions rather than ranking the methods by error. The error definitions are not uniform and the figures should not be differenced against one another: the embedded methods report a percentage of the reading or of the monitored prestress under a calibrated model, whereas the figure reported here is a percentage of full scale obtained under cross-validation in which a complete tensioning round was withheld. For the through-type approach [21] no numerical value is entered, because its target quantity, denominator and validation protocol were not confirmed against the original article.

## Data Availability

The data presented in this study are available on request from the corresponding author. The data are not publicly available because they form part of an ongoing government-funded research program.

## Abbreviations

DAQ	Data acquisition
EM	Elasto-magnetic
FS	Full scale
GPR	Gaussian process regression
LSBoost	Least-squares gradient boosting of regression trees
MFL	Magnetic flux leakage
NDT	Non-destructive testing
NTC	Negative temperature coefficient
PCB	Printed circuit board
PLS	Partial least squares regression
PSC	Prestressed concrete
RMSE	Root-mean-square error
